# Phthalimide‐Based High Mobility Polymer Semiconductors for Efficient Nonfullerene Solar Cells with Power Conversion Efficiencies over 13%

**DOI:** 10.1002/advs.201801743

**Published:** 2018-12-12

**Authors:** Jianwei Yu, Peng Chen, Chang Woo Koh, Hang Wang, Kun Yang, Xin Zhou, Bin Liu, Qiaogan Liao, Jianhua Chen, Huiliang Sun, Han Young Woo, Shiming Zhang, Xugang Guo

**Affiliations:** ^1^ Department of Materials Science and Engineering and The Shenzhen Key Laboratory for Printed Organic Electronics Southern University of Science and Technology No. 1088, Xueyuan Road Shenzhen Guangdong 518055 China; ^2^ Key Laboratory of Flexible Electronics (KLOFE) and Institute of Advanced Materials (IAM) Jiangsu National Synergetic Innovation Center for Advanced Materials (SICAM) Nanjing Tech University (NanjingTech) 30 South Puzhu Road Nanjing 211816 China; ^3^ Research Institute for Natural Sciences Department of Chemistry Korea University Seoul 136‐713 South Korea

**Keywords:** difluorobenzothiadiazole, high mobility polymers, high power conversion efficiencies, nonfullerene polymer solar cells, phthalimide

## Abstract

Highly efficient nonfullerene polymer solar cells (PSCs) are developed based on two new phthalimide‐based polymers phthalimide‐difluorobenzothiadiazole (PhI‐ffBT) and fluorinated phthalimide‐ffBT (ffPhI‐ffBT). Compared to all high‐performance polymers reported, which are exclusively based on benzo[1,2‐*b*:4,5‐*b*′]dithiophene (BDT), both PhI‐ffBT and ffPhI‐ffBT are BDT‐free and feature a D‐A_1_‐D‐A_2_ type backbone. Incorporating a second acceptor unit difluorobenzothiadiazole leads to polymers with low‐lying highest occupied molecular orbital levels (≈−5.6 eV) and a complementary absorption with the narrow bandgap nonfullerene acceptor IT‐4F. Moreover, these BDT‐free polymers show substantially higher hole mobilities than BDT‐based polymers, which are beneficial to charge transport and extraction in solar cells. The PSCs containing difluorinated phthalimide‐based polymer ffPhI‐ffBT achieve a substantial PCE of 12.74% and a large *V*_oc_ of 0.94 V, and the PSCs containing phthalimide‐based polymer PhI‐ffBT show a further increased PCE of 13.31% with a higher *J*_sc_ of 19.41 mA cm^−2^ and a larger fill factor of 0.76. The 13.31% PCE is the highest value except the widely studied BDT‐based polymers and is also the highest among all benzothiadiazole‐based polymers. The results demonstrate that phthalimides are excellent building blocks for enabling donor polymers with the state‐of‐the‐art performance in nonfullerene PSCs and the BDT is not necessary for constructing such donor polymers.

## 1 Introduction

Bulk heterojunction (BHJ) polymer solar cells (PSCs) have attracted substantial attention in the last two decades owing to their unique advantages of being flexible, light‐weight, low‐cost, and solution‐processable, enabling device fabrication in a large area via a high‐throughput roll‐to‐roll fashion.^1^, ^2^, ^3^, ^4^ BHJ PSCs typically consist of a p‐type polymer as electron donor material and an n‐type semiconductor as electron acceptor, which used to be fullerene‐based derivative.^2^, ^5^ Thanks to the development of high‐performance polymer donors, the current state‐of‐the‐art PCEs of single‐layer fullerene‐based PSCs have reached 11.7%.^6^ However, such PSCs are plagued by the intrinsic drawbacks of fullerene derivatives including high synthetic cost, weak visible light absorption, limited tunability of molecular structures and energy levels of frontier molecular orbitals (FMOs), and poor stability of blend film morphologies.^7^, ^8^ Recently, substantial efforts have been devoted to the development of nonfullerene‐based electron acceptors,^3^, ^9^, ^10^, ^11^, ^12^, ^13^, ^14^ particularly the fused‐ring electron acceptors (FREAs),^15^, ^16^, ^17^, ^18^ which hold various merits including strong absorption in the visible to near‐infrared region, easy tunability of electronic structures and FMO energy levels through molecular engineering, as well as improved blend morphology stabilities. As a result, a great number of FREAs with various backbones, side chain substituents, and terminal groups, have been synthesized in the past several years and led to the maximum PCE > 14% in single‐layer solar cells,^19^, ^20^, ^21^ demonstrating unprecedented potentials of nonfullerene acceptors for enabling high‐performance photovoltaic devices for practical applications.^22^, ^23^

Along with the advancement of novel FREAs, developing appropriate donor polymers having good compatibility with acceptor materials is equally critical for attaining high‐performance nonfullerene PSCs.^24^ Ideally, a suitable donor polymer should hold complementary absorption with that of the narrow bandgap FREAs, thus maximizing light harvesting and exciton generation. Additionally, the FMO energy levels of the polymer donors should be appropriately aligned in order to match well with those of the FREAs for implementing efficient exciton dissociation and minimizing photon energy loss (*E*_loss_ = *E*_g_ − e*V*_oc_) in the corresponding devices.^25^ Moreover, for achieving efficient charge transport and extraction in the polymer:FREA blends, a high and balanced hole/electron mobility and a favorable blend film morphology should also be granted, on that account large short‐current densities (*J*_sc_) and high fill factors (FFs) can be envisioned.^19^, ^26^, ^27^ With these considerations, a plenty of donor polymers have been designed and synthesized to match with the new emerging FREAs, showing promising device performances in nonfullerene PSCs.

**Figure 1:**
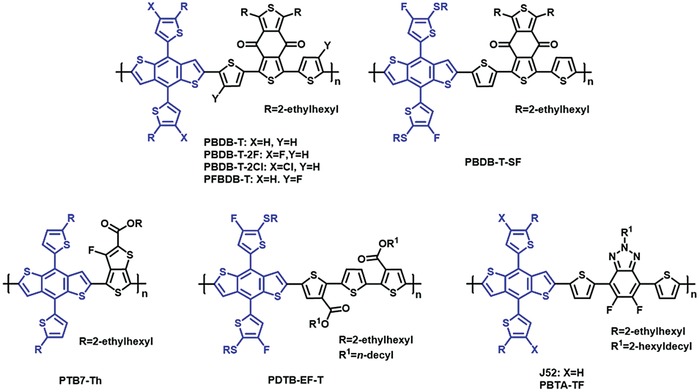
Chemical structures of the representative donor polymers reported in literatures with power conversion efficiencies >13% in single‐layer binary solar cells. All these polymers are based on benzo[1,2‐*b*:4,5‐*b*′]dithiophene, which is marked in blue.

A great number of donor polymers have been developed to date, but a very few of them have showed PCEs over 13% in the single‐junction binary solar cells.^19^, ^20^, ^28^, ^29^, ^30^, ^31^, ^32^, ^33^, ^34^, ^35^, ^36^, ^37^, ^38^, ^39^, ^40^ To the best of our knowledge, all these high‐performance donor polymers are alternating donor–acceptor (D‐A) type copolymers, which are exclusively based on benzo[1,2‐*b*:4,5‐*b*′]dithiophene (BDT) donor unit^41^ copolymerized with a few acceptor counits, such as 5,6‐difluoro‐2‐alkyl‐2*H*‐benzo[*d*][1,2,3]triazole (FTAZ),^42^ benzo[1,2‐*c*:4,5‐*c*′]‐dithiophene‐4,8‐dione (BDD)^43^ etc.^37^ (**Figure** **1**). Moreover, due to the high aromatic resonance energy of benzene moiety in BDT, the π‐orbitals of BDT show a high degree of localization,^44^ which is not beneficial to intramolecular charge carrier delocalization. Therefore, the BDT‐based polymers typically show low motilities in neat films, as revealed by mobility measurement in organic thin‐film transistors (OTFTs).^41^, ^45^ By replacement of BDT with oligothiophenes, the bithiophene imide‐oligothiophene copolymers show greatly improved charge carrier mobilities, yielding much higher *J*_sc_ and FFs in solar cells.^27^, ^46^ In these senses, it is highly imperative to develop donor polymers based on new building blocks with distinct structure motifs to enrich the materials diversity, improve the charge transport property, and further enhance the PSC efficiency, which, additionally, provide new platforms for studying the fundamental materials structure–property correlations.^47^, ^48^

**Figure 2:**
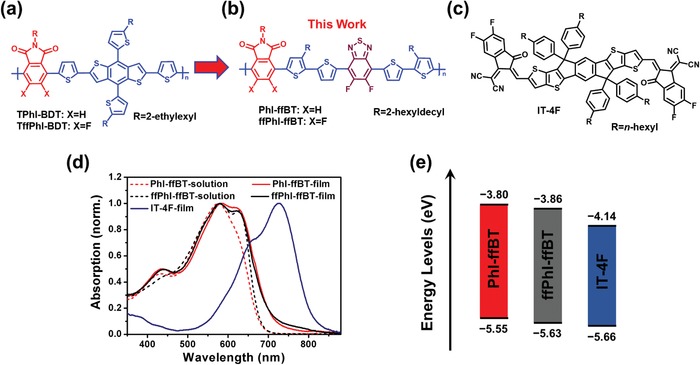
Chemical structures of a) the previously reported phthalimide‐based D‐A type wide bandgap donor polymers, which contain benzo[1,2‐*b*:4,5‐*b*′]dithiophene (BDT) in backbone and b) the phthalimide‐based BDT‐free D‐A_1_‐D‐A_2_ type medium bandgap donor polymers. The introduction of the second stronger acceptor difluorobenzothiadiazole leads to optimized polymer optoelectronic properties, and c) the nonfullerene acceptor IT‐4F; d) absorption spectra of PhI‐ffBT and ffPhI‐ffBT in chlorobenzene (10^−5^ m) and thin films spin‐coated from chlorobenzene solution (5 mg mL^−1^) together with the ITIC film absorption; e) FMO energy level diagram of PhI‐ffBT, ffPhI‐ffBT, and IT‐4F.

Phthalimide (PhI), an imide‐functionalized benzene, has been much less explored in the field of organic electronics,^49^, ^50^, ^51^ in comparison to other imide‐functionalized arenes.^52^, ^53^, ^54^, ^55^ We first introduced it to high‐mobility polymer semiconductors^49^ and recently developed a series of wide bandgap D‐A type copolymers TPhI‐BDT based on PhI and TffPhI‐BDT (**Figure** **2**a) based on fluorinated phthalimide (ffPhI) for nonfullerene PSCs, by copolymerizing it with BDT. The solar cells achieved the highest PCE of 9.48% with a *J*_sc_ of 15.92 mA cm^−2^ and a FF of 63.9%, when blended with a narrow bandgap IDIC, suggesting the great promises of PhI‐based polymers for enabling efficient nonfullerene PSCs.^56^ Nevertheless, the *J*_sc_ and the FF of these PhI‐based PSCs are relatively small due to the unsatisfactory absorption, limited and unbalanced charge carrier mobilities, and unfavorable blend film morphology, which indicate that further materials optimizations are needed in order to fully explore the potentials of PhI‐based polymer semiconductors.

In this context, we introduced a second acceptor unit difluorobenzothiadiazole (ffBT) into polymer backbones and developed two PhI‐based donor‐acceptor #1‐donor‐acceptor #2 (D‐A_1_‐D‐A_2_) type donor polymers PhI‐ffBT and ffPhI‐ffBT without using BDT (Figure 2b), showing fine‐tuned optoelectronic structures for highly efficient nonfullerene PSCs.^57^ Compared to the PhI‐based alternating D‐A type polymer analogues, the incorporation of the stronger electron‐withdrawing ffBT unit (vs PhI) enables an intensified intramolecular charge transfer and leads to a broadened and redshifted absorption approaching 700 nm,^58^ thus yielding a more complementary absorption with a FREA molecule IT‐4F^32^ and a high *J*_sc_ > 19.0 mA cm^−2^ in the PSCs. Moreover, the stronger acceptor ffBT optimizes the electronic properties of PhI‐based polymers, resulting in downshifted highest occupied molecular orbital (HOMO) levels^59^ of −5.55 and −5.63 eV and hence yielding high *V*_oc_ > 0.9 V with small energy losses (*E*_loss_, ≈0.60 eV). In addition, as an excellent building block for high mobility polymers,^58^, ^60^ the incorporation of ffBT should promote the self‐assembly of the resulting polymers, and the elimination of BDT should facilitate the charger carrier delocalization, therefore these D‐A_1_‐D‐A_2_ type polymers show a more crystalline structure and greatly increased hole mobilities of 0.6–0.9 cm^2^ V^−1^ s^−1^ in OTFTs, which eventually yield a high FF of 0.76 in solar cells. Benefitting from these distinctive advantages, ffPhI‐ffBT:IT‐4F‐based PSCs exhibited a large *V*_oc_ of 0.94 V and a relatively small energy loss (*E*_loss_) of 0.58 eV with a PCE of 12.74%. Compared to the ffPhI‐ffBT‐based devices, a further increased PCE of 13.31% was achieved for the PhI‐ffBT:IT‐4F‐based PSCs with an *E*_loss_ of 0.61 eV attributed to the improved film morphology and higher charge carrier mobilities of the blend film. As the first example of PhI‐based polymers with a PCE > 13%, the results demonstrate the great potentials of phthalimide for enabling donor polymers with optimized optoelectronic properties and film morphologies to achieve remarkable performance in nonfullerene PSCs.

## 2 Results and Discussion

### 2.1 Synthesis of Polymers

**Scheme 1:**
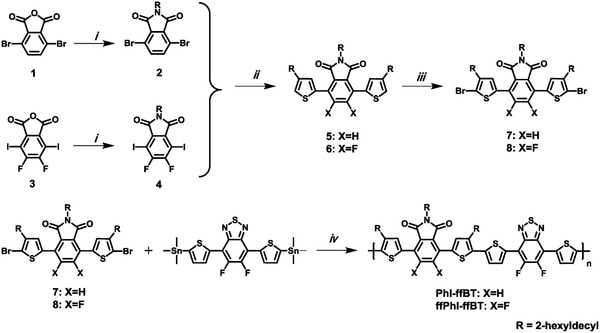
Synthetic routes to the phthalimide‐based monomers and their corresponding polymer semiconductors. Reagents and condition: (i) 2‐hexyldecan‐1‐amine, AcOH; (ii) tributyl(4‐(2‐hexyldecyl)thiophen‐2‐yl)stannane, Pd(PPh_3_)_2_Cl_2_; (iii) NBS, DMF; (iv) Pd_2_(dba)_3_, P(*o*‐tolyl)_3_, toluene.

The synthetic routes to these two phthalimide‐based polymers PhI‐ffBT and ffPhI‐ffBT are straightforward (**Scheme** **1**), and the synthetic details are included in the Supporting Information. Briefly, compound **2** can be readily prepared through the imidization of the commercially available 4,7‐dibromoisobenzofuran‐1,3‐dione **1** with a high yield. It was then coupled with tributyl(4‐(2‐hexyldecyl)thiophen‐2‐yl)stannane through Stille‐coupling reaction, yielding the thiophene flanked **5**. Compound **5** was then brominated using *N*‐bromosuccinimide (NBS) to afford the monomer **7**. Difluorinated monomer **8** was prepared by following the similar synthetic route to monomer **7** from the anhydride **3**. Notably, due to the lack of solubilizing chains on the ffBT moiety, here the large branched alkyl chain 2‐hexyldecyl was introduced into the monomers **7** and **8** to ensure the sufficient solubility of the resulting polymers. Finally, the two phthalimide‐based polymer semiconductors, PhI‐ffBT and ffPhI‐ffBT, were prepared via Stille coupling‐based polycondensation under microwave irradiation. After polymerizations, both polymers exhibit good solubility in common organic solvents, attributed to the solubilizing chains on the imide groups, which have been approved as an additional advantage of imide‐functionalized arenes.^52^ As shown in **Table** **1**, the number‐average molecular weight (*M*_n_) of PhI‐ffBT and ffPhI‐ffBT is 36 and 58 kDa with a polydispersity index (PDI: *M*_w_/*M*_n_) of 1.5 and 1.2, respectively, measured by high‐temperature gel permeation chromatography (GPC) at 150 °C. The decomposition temperatures (*T*_d_), defined as the temperature with a 5% weight loss, of PhI‐ffBT and ffPhI‐ffBT are ≈440 °C, indicative of their good thermal stability for device optimization (Figure S1a, Supporting Information). Differential scanning calorimetry (DSC) reveals that both PhI‐ffBT and ffPhI‐ffBT exhibit distinctive thermal transition peaks, indicating a high degree of crystallinity of both polymers (Figure S1b, Supporting Information). These results greatly differ from those of BDT‐based polymer analogues showing featureless DSC profiles,^56^ which indicates the improved materials crystallinity of polymers PhI‐ffBT and ffPhI‐ffBT by eliminating BDT and incorporating in polymer backbones.

**Table 1:** Molecular weight, optical characteristics, and electrochemical property of the phthalimide‐based polymer semiconductors PhI‐ffBT and ffPhI‐ffBT

Polymer	*M*_n_ a) [kDa]	PDI	*T*_d_ b) [°C]	λ_max_ c) (sol) [nm]	λ_max_ d) (film) [nm]	λ_onset_ d) (film) [nm]	*E*_HOMO_ e) [eV]	*E*_LUMO_ f) [eV]	*E*_g_^opt^ g) [eV]
PhI‐ffBT	36	1.5	440	575	583	708	−5.55	−3.80	1.75
ffPhI‐ffBT	58	1.2	439	580	580	700	−5.63	−3.86	1.77

^a)^ GPC using 1,2,4‐trichlorobenzene as the eluent at 150 °C versus polystyrene standards

^b)^ Temperature with a 5% weight loss

^c)^ From chlorobenzene solution (10^−5^ m)

^d)^ From pristine films spin‐casted from chlorobenzene solutions (5 mg mL^−1^)

^e)^ *E*_HOMO_ = −e(*E*_ox_^onset^ + 4.80) eV, and *E*_ox_^onset^ determined from CV versus Fc/Fc^+^ standard

^f)^ *E*_LUMO_ = *E*_HOMO_ + *E*_g_^opt^

^g)^ Estimated from polymer film absorption onsets: *E*_g_^opt^ = 1240/λ_onset_ eV.

### 2.2 Optical and Electrochemical Properties of Polymers

Figure 2d illustrates the UV–vis absorption spectra of PhI‐ffBT and ffPhI‐ffBT, and Table 1 summarizes their corresponding absorption parameters. From solution to film, both polymers exhibit minimal bathochromic shifts of <≈8 nm in terms of absorption peaks (λ_max_), which together with their structured absorption profile indicate their strong aggregation character in solution. Compared to PhI‐ffBT, the fluorinated phthalimide‐based ffPhI‐ffBT shows a well‐resolved absorption shoulder in solution, indicative of its stronger aggregation. In addition, both PhI‐ffBT and ffPhI‐ffBT exhibit distinctive temperature‐dependent absorption as illustrated in Figure S3 (Supporting Information), which further confirms the strong interpolymer chain interactions in solutions. Both PhI‐ffBT and ffPhI‐ffBT films show a pronounced absorption shoulder at 615 and 620 nm, respectively, implying their ordered structure in solid state. The strong aggregation property of PhI‐ffBT and ffPhI‐ffBT is mainly attributed to their planar backbones and intense intermolecular attractions enabled by intra‐ and/or intermolecular F···H and F···S interactions.^61^ The optical bandgaps (*E*_g_^opt^) derived from film absorption onsets are 1.75 and 1.77 eV for PhI‐ffBT and ffPhI‐ffBT, respectively, which are ≈0.25 eV smaller than those of the BDT‐based polymers,^56^ corroborating a more delocalized π‐conjugation length by eliminating the BDT moiety. In addition, the absorption coefficients of polymer films were measured, and it was found that the ffPhI‐based polymers TffPhI‐BDT and ffPhI‐ffBT show higher coefficients than PhI‐based analogue polymers TPhI‐BDT and PhI‐ffBT (Figure S4, Supporting Information). Among them, the BDT‐based polymer TffPhI‐BDT exhibits the highest absorption coefficient. However, the BDT‐free polymers PhI‐ffBT and ffPhI‐ffBT show distinctly redshifted absorption (≈100 nm), which results in a perfectly complementary absorption with that of narrow bandgap nonfullerene acceptor, IT‐4F (Figure 2c), leading to optimized light harvesting in PSC devices.

The FMO energy levels of the polymer films were investigated using cyclic voltammetry (CV). Based on the oxidation onset (*E*_ox_^onset^), the equation *E*_HOMO_ = −*e*(*E*_ox_^onset^ + 4.80) eV was used to calculate the polymer HOMO levels. As shown in Figure S2 (Supporting Information), the HOMO levels (*E*_HOMO_) are found to be −5.55 and −5.63 eV for PhI‐ffBT and ffPhI‐ffBT, respectively. Such low‐lying HOMOs should lead to large *V*_oc_ in solar cells. Compared to that of PhI‐ffBT, the deeper‐positioned HOMO of ffPhI‐ffBT is attributed to the additional electron‐withdrawing F atoms on the phthalimide moiety, which should be further beneficial to *V*_oc_ in PSCs. The LUMO levels (*E*_LUMO_) of PhI‐ffBT and ffPhI‐ffBT are −3.80 and −3.86 eV, respectively, calculated from their *E*_HOMO_ and *E*_g_^opt^ using the equation of *E*_LUMO_ = *E*_HOMO_ + *E*_g_^opt^. The Δ*E*_HOMO_ between PhI‐ffBT/ffPhI‐ffBT and IT‐4F (HOMO = −5.66 eV) is only 0.11/0.03 eV. Such small HOMO offset should be beneficial for reducing the *E*_loss_ in nonfullerene PSCs.^62^ Density functional theory (DFT)‐based calculations were also carried out to investigate the molecular geometry and electronic properties of these phthalimide‐based polymers using a hybrid B3LYP correlation function and 6‐31G(d,p) basis set. As shown in Figure S5 (Supporting Information), both polymers feature a high degree of backbone planarity, which should be conducive to polymer chain packing and charge transport. The computation results reveal that the *E*_LUMO_/*E*_HOMO_ (−2.92/−5.07 eV) of the ffPhI‐ffBT is lower than those (−2.90/−4.99 eV) of PhI‐ffBT (Figure S6, Supporting Information), which are consistent well with the trend of the experimental results.

### 2.3 Organic Thin‐Film Transistor and Polymer Solar Cell Performance

**Figure 3:**
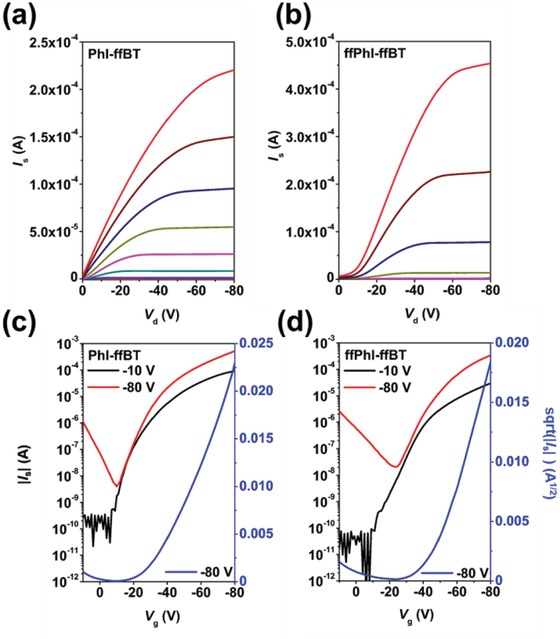
Top‐gate/bottom‐contact (TG/BC) OTFT a,b) output and c,d) transfer characteristics of (a,c) PhI‐ffBT and (b,d) ffPhI‐ffBT. The gate voltage range is 0 to −80 V with −10 V intervals in the output plots.

In order to investigate the charge transport properties of these phthalimide‐based polymers PhI‐ffBT and ffPhI‐ffBT, top‐gate/bottom‐contact (TG/BC) OTFTs were fabricated with a device architecture of glass/Au/polymer/CYTOP/Al, where a 380 nm thick amorphous fluoropolymer CYTOP (Asahi Glass Co., Ltd.) with an areal capacitance of 4.54 nF cm^−2^ was used as the gate dielectric layer. Both PhI‐ffBT and ffPhI‐ffBT show the p‐type dominating transport characteristics (**Figure** **3**) and the OTFTs annealed at 160 °C yield the optimal performance with the highest hole mobility (*µ*_h,OTFT_) of 0.63 and 0.93 cm^2^ V^−1^ s^−1^ in the saturated regime, respectively. In the linear regime, the OTFTs show lower device performance with the highest *µ*_h,OTFT_ of 0.21 and 0.23 cm^2^ V^−1^ s^−1^ for PhI‐ffBT and ffPhI‐ffBT (**Table** **2**), respectively. Such mobility reduction from saturated to linear regime is due to the contact resistance effect.^63^ In addition, only a slight hysteresis is observed in the transfer curves for all these phthalimide‐based polymers (Figure S7, Supporting Information), resulting in a small difference between the mobilities extracted from the forward and reverse sweeps (Figure S9, Supporting Information). In fact, the mobilities reported are calculated using the average slope in the −70 to −80 V region, and only the forward sweep data were used to ensure their reliability. On the basis of the OTFT output characteristics and the threshold voltages (*V*_T_), the difluorinated phthalimide‐based polymer ffPhI‐ffBT shows a larger contact resistance due to its lower‐positioned HOMO energy level, which results in a higher hole injection barrier with the Au source/drain electrodes. Compared to the BDT containing polymer analogues TPhI‐BDT and TffPhI‐BDT, showing a *µ*_h,OTFT_ of ≤≈10^−2^ cm^2^ V^−1^ s^−1^ (Figure S8 and Table S2, Supporting Information), these BDT‐free polymers PhI‐ffBT and ffPhI‐ffBT exhibit a distinctly improved mobility by 1–2 orders of magnitude in OTFT devices, which demonstrates the superiority of these D‐A_1_‐D‐A_2_ type polymers on promoting charge transport (vs the BDT‐based polymers).

**Table 2:** Top‐gate/bottom‐contact (TG/BC) OTFT performance parameters of polymers PhI‐ffBT and ffPhI‐ffBT fabricated under the optimal conditions

Polymer	*T*_a_ [°C]	*µ*_h,lin_ a) [cm^2^ V^−1^ s^−1^]	*µ*_h,sat_ a) [cm^2^ V^−1^ s^−1^]	*V*_T_ b) [V]	*I*_on_/*I*_off_ b)
PhI‐ffBT	160	0.21 (0.19)	0.63 (0.51)	−33	10^5^
ffPhI‐ffBT	160	0.23 (0.17)	0.93 (0.72)	−40	10^4^

^a)^ Maximum mobilities with average values from at least five devices shown in parentheses

^b)^ Average values shown.

**Figure 4:**
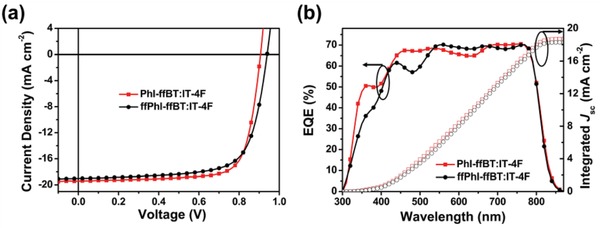
a) *J−V* characteristics and b) EQE spectra of the optimized conventional PSC devices with the phthalimide‐based polymer as the donor material and nonfullerene IT‐4F as the acceptor material.

PSCs with a conventional device structure of ITO/PEDOT:PSS/active layer/PDINO/Al with a device area of 4.5 mm^2^ were fabricated to investigate the photovoltaic properties of PhI‐ffBT and ffPhI‐ffBT, where poly(3,4‐ethylenedioxythiophene):polystyrene sulfonate (PEDOT:PSS) and a perylene diimide derivative with amino *N*‐oxide terminal substituent (PDINO) serve as the hole and electron transporting layer, respectively. IT‐4F^32^ was chosen as the acceptor material due to its complementary absorption and well‐matched energy levels with these PhI‐based polymer donor materials. Various device fabrication conditions including varying processing additive volume ratios, casting solvents, polymer:IT‐4F weight ratios, thermal annealing temperatures, and device structures were systematically tested in order to optimize the PSC performance (Tables S3–S7, Supporting Information). **Figure** **4**a shows the current density–voltage (*J*–*V*) curves of the optimized solar cells, and **Table** **3** summarizes the corresponding performance parameters. As expected from the low‐lying polymer HOMO levels, both PhI‐ffBT and ffPhI‐ffBT cells exhibit large *V*_oc_ ≥ 0.90 V. The additional F atoms on the phthalimide lead to a deeper HOMO level for polymer ffPhI‐ffBT, which is translated to a smaller *E*_loss_ (0.58 eV) and a larger *V*_oc_ (0.94 V) than those (*E*_loss_ = 0.61 eV; *V*_oc_ = 0.91 V) of PhI‐ffBT in PSCs. For ffPhI‐ffBT‐based devices, a highest PCE of 12.74% with a *J*_sc_ of 19.01 mA cm^−2^ and a FF of 0.71 was obtained using ffPhI‐ffBT:IT‐4F ratio of 1.2:1, 0.6% (volume ratio) 1,8‐diiodooctane (DIO) additive, and a thermal treatment at 125 °C for 5 min. For PhI‐ffBT‐based devices, although a slightly smaller *V*_oc_ of 0.91 V was attained, an enhanced PCE up to 13.31% was realized, mainly attributed to the largely increased FF of 0.76 (vs 0.71 for the ffPhI‐ffBT‐based cells). In addition, the PSCs with a larger effective area of 10 mm^2^ are also fabricated, which show a slightly reduced PCE of 12.93 and 12.24% for PhI‐ffBT‐and ffPhI‐ffBT‐based devices, respectively (Table S8 and Figure S11, Supporting Information). Such drop of solar cell performances is mainly attributed to the decreased FFs as a result of increased bimolecular recombination, which has been observed in many other solar cells.^28^, ^64^, ^65^, ^66^

**Table 3:** Photovoltaic performance parameters of ffPhI‐ffBT and PhI‐ffBT‐based nonfullerene solar cells with an active area of 4.5 mm^2^

Polymer	*V*_oc_ a) [V]	*J*_sc_ a) [mA cm^−2^]	*J*_cal_ b) [mA cm^−2^]	FFa)	PCEa) [%]
PhI‐ffBT	0.91 (0.90 ± 0.01)	19.41 (19.08 ± 0.27)	18.69	0.76 (0.75 ± 0.01)	13.31 (12.92 ± 0.23)
ffPhI‐ffBT	0.94 (0.94 ± 0.003)	19.01 (18.84 ± 0.18)	18.28	0.71 (0.71 ± 0.01)	12.74 (12.42 ± 0.18)

^a)^ The maximum values with the average values and standard deviations based on 15 devices shown in parenthesis

^b)^ Integrated from EQE curves.

Inverted PSCs with a structure of ITO/ZnO/active layer/MoO_3_/Ag were also constructed to further evaluate the photovoltaic performance of both polymers. Compared to that of conventional PSCs, a similar performance trend was observed but with slightly lower PCE values, that is, 11.60 and 12.14% for ffPhI‐ffBT and PhI‐ffBT‐based PSCs, respectively (Table S7 and Figure S10a, Supporting Information). These results demonstrate the high efficacy of both phthalimide and difluorinated phthalimide for enabling polymer semiconductors with remarkable device performance in nonfullerene‐based PSCs. Please note that in comparison to the previously reported BDT‐based polymer analogues,^56^ these BDT‐free polymers show substantially improved *J*_sc_ and FFs, which can be partially attributed to their improved charge transport properties as revealed by mobility measurement using both OTFTs and space charge limited current (SCLC) method (vide infra).

Figure 4b shows the external quantum efficiency (EQE) spectra of the optimized PSC devices. Benefitting from the broad and complementary absorption as well as efficient photo‐to‐current response, high integrated currents (*J*_cal_) of 18.69 and 18.28 mA cm^−2^ were obtained for the PhI‐ffBT‐ and ffPhI‐ffBT‐based devices, respectively. Compared to the ffPhI‐ffBT cells, the PhI‐ffBT cells displayed more efficient photoresponse in the short wavelength region, resulting in a slightly higher *J*_cal_. The mismatch ratios between integrated *J*_cal_ from EQE curves and *J*_sc_ from *J*–*V* curves are <4%, showing good reliability of the photovoltaic performance of these PSCs.

### 2.4 Charge Generation, Transfer, and Transport Characteristics

**Figure 5:**
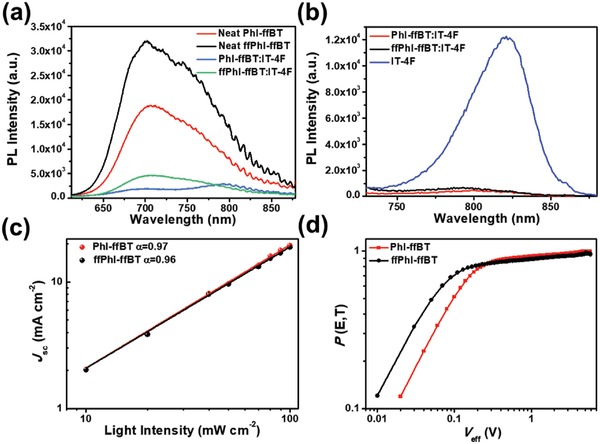
PL spectra of a) neat polymer films and blend films excited at 550 nm, and b) neat IT‐4F and blend films excited at 700 nm; c) the light intensity‐dependent *J*–*V* characteristics of the PhI‐ffBT:IT‐4F and ffPhI‐ffBT:IT‐4F‐based PSCs; d) charge collection probability (*P*(E, T)) as a function of effective bias (*V*_eff_) in PhI‐ffBT:IT‐4F and ffPhI‐ffBT:IT‐4F‐based PSCs.

The photoluminescence (PL) measurements were carried out to evaluate the exciton dissociation efficiencies at the donor:acceptor interfaces, and the spectra excited at different wavelengths are shown in **Figure** **5**a,b. In comparison to that (86%) of the ffPhI‐ffBT:IT‐4F blend, the PhI‐ffBT:IT‐4F blend exhibits a higher PL quenching efficiency (PLQE) of 90% when excited at 550 nm, suggesting more efficient electron transfer from the excited donor polymer to IT‐4F in the PhI‐ffBT:IT‐4F blends versus the ffPhI‐ffBT:IT‐4F blends. Then, both pure IT‐4F and blend films were excited at 700 nm to evaluate the hole transfer efficiency from the excited IT‐4F to the donor polymer. Even though very small Δ*E*_HOMO_, 0.11 eV for PhI‐ffBT:IT‐4F and 0.03 eV ffPhI‐ffBT:IT‐4F were observed in the CV measurements, these small Δ*E*_HOMO_ could still provide sufficient driving force for realizing efficient hole transfer from excited IT‐4F to donor polymer as evidenced by the high PLQEs, 95% for ffPhI‐ffBT blend films and 96% for PhI‐ffBT blend films.

The charge carrier mobilities of the blend films were further investigated using SCLC method (Figure S12 and Table S9, Supporting Information). The fabrication details for hole‐only and electron‐only devices are included in the Supporting Information. In this study, all the SCLC mobility values are extracted from the trap‐free region at high voltage (2–4 V for all devices) which follows the standard SCLC equation (*J* ≈ *V* ^2^), and the experimental *J–V* curve can match well over a large voltage range, indicating that the extracted SCLC mobility reflects the charge transport property of the blend active layer. The optimized PhI‐ffBT:IT‐4F blend displayed a *µ*_h,SCLC_ and *µ*_e,SCLC_ of 1.42 × 10^−3^ and 5.49 × 10^−4^ cm^2^ V^−1^ s^−1^, respectively, both of which are much higher than those (*µ*_h,SCLC_ of 5.61 × 10^−4^ cm^2^ V^−1^ s^−1^ and a *µ*_e,SCLC_ of 2.21 × 10^−4^ cm^2^ V^−1^ s^−1^) of ffPhI‐ffBT‐based blend film. The increased mobilities of PhI‐ffBT:IT‐4F film lead to higher FF of PhI‐ffBT‐based PSCs. In good agreement with the hole mobilities measured from OTFT devices, the *µ*_h,SCLC_ (≈1 × 10^−3^ cm^2^ V^−1^ s^−1^) of these BDT‐free polymers are substantially larger than those (≈7 × 10^−5^ cm^2^ V^−1^ s^−1^) of BDT‐based polymer analogues TPhI‐BDT and TffPhI‐BDT,^56^ yielding much higher *J*_sc_ and FFs in solar cells. To gain deeper insights into the PSC performance, the light intensity‐dependent *J*–*V* characteristics of the ffPhI‐ffBT:IT‐4F and PhI‐ffBT:IT‐4F devices were also measured to shed light on the bimolecular charge recombination, which plays a critical role in determining FF and *J*_sc_. Figure 5c illustrates the *J*_sc_ as a function of spectral irradiance *P* (*J*∝*P^α^*, where α is the recombination parameter) of both polymer‐based PSCs under the optimized conditions. Both PhI‐ffBT and ffPhI‐ffBT‐based cells show high α values approaching unity (0.96 and 0.97 for ffPhI‐ffBT and PhI‐ffBT‐based cells, respectively), indicating the substantially suppressed bimolecular recombination of charge carriers in blend films. The exciton dissociation probabilities (*P*(E, T)) were further measured.^67^, ^68^, ^69^ As shown in Figure 5d, the *P*(E, T) of PhI‐ffBT and ffPhI‐ffBT‐based active layers at short‐circuit condition is 93 and 88%, respectively, indicating more efficient exciton dissociation and charge collection probability for PhI‐ffBT‐based solar cells. The results are well correlated with the PL quenching efficiencies and film morphological characters as revealed by atomic force microscopy (AFM) (vide infra).

### 2.5 Film Morphologies and Their Correlations to Device Performance

**Figure 6:**
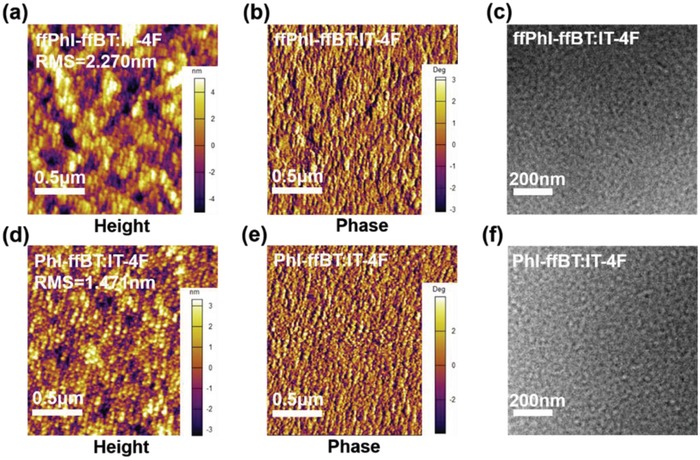
Tapping‐mode AFM height and phase images for a,b) ffPhI‐ffBT:IT‐4F and d,e) PhI‐ffBT:IT‐4F blend films; c,f) TEM images for ffPhI‐ffBT:IT‐4F and PhI‐ffBT:IT‐4F blend films.

The polymer:IT‐4F film morphologies were investigated by utilizing AFM and transmission electron microscopy (TEM) measurements (**Figure** **6**, Figures S13–S15 and Table S10, Supporting Information). As shown in Figure 6a,d, the root‐mean‐square (RMS) roughness of PhI‐ffBT:IT‐4F and ffPhI‐ffBT:IT‐4F blend films prepared under the optimized device fabrication conditions is 1.47 and 2.27 nm, respectively. The smoother surface morphology of PhI‐ffBT:IT‐4F film likely indicates a better miscibility between polymer donor PhI‐ffBT and the nonfullerene acceptor IT‐4F, which helps achieve a higher *J*_sc_ and FF. As shown by TEM images (Figure 6c,f), similar well‐defined phase separations at nanoscale with a highly interpenetrating and bicontinuous network were clearly observed for the blend films of both polymers, which are beneficial for realizing efficient exciton dissociation and charge carrier collection.^70^

**Figure 7:**
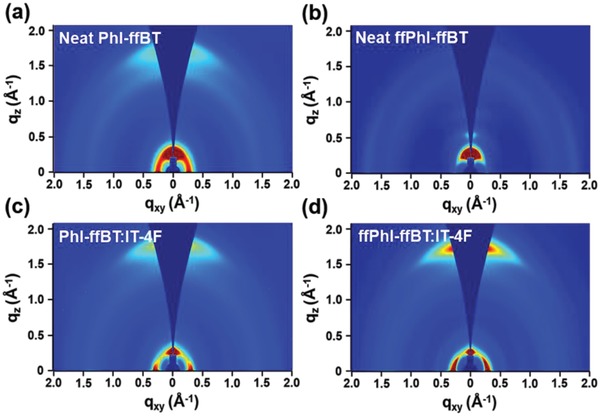
GIWAXS images of a,b) neat polymer films and c,d) polymer:IT‐4F blend films after the thermal treatment.

To further illustrate the morphology–performance correlations, 2D grazing incidence wide angle X‐ray scattering (2D GIWAXS) measurement was carried out. **Figure** **7** and Figure S16 (Supporting Information) show the 2D GIWAXS patterns and the corresponding line‐cut profiles of in‐plane (IP) and out‐of‐plane (OOP) directions of PhI‐ffBT, ffPhI‐ffBT, PhI‐ffBT:IT‐4F, and ffPhI‐ffBT:IT‐4F films. As shown in Figure 7a,b, ffPhI‐ffBT neat film displays a predominant edge‐on orientation, showing clear lamellar scatterings up to (300) in the OOP direction together with a (010) peak in the IP direction. On the contrary, the neat PhI‐ffBT polymer adopts a bimodal orientation (both face‐on and edge‐on) with a (100) lamellar peak in both IP and OOP directions, but the intense (010) diffraction was observed (*d*‐spacing 3.67 Å) in OOP direction. Hence, the fluorination of phthalimide moiety leads to an increased edge‐on orientation of polymer ffPhI‐ffBT versus polymer PhI‐ffBT, and such phenomena was also observed in other materials.^71^, ^72^ It is interesting to note that fluorination or the increased degree of multi‐fluorination can show an opposite effect, promoting face‐on orientation of polymer chains.^71^, ^72^ Even for the same polymer, the molecular weight^69^ and the thermal treatment^73^ can dramatically alter chain orientation. All these observations reflect the complexity of various interactions, including interpolymer chain interaction (aggregation), interaction between polymer backbone with substrate, and interaction between polymer side chain with substrate, on determining polymer orientation.

On the basis of (100) diffractions, PhI‐ffBT shows a lower degree of crystallinity than ffPhI‐ffBT, which is likely attributed to the extra intra‐ and/or intermolecular F···H and F···S interactions in the fluorinated phthalimide‐based polymer ffPhI‐ffBT. As a result, the higher crystallinity and predominant edge‐on polymer backbone orientation of ffPhI‐ffBT yields its higher hole mobility in OTFT devices (vs PhI‐ffBT). For the blend films after thermal treatment, both ffPhI‐ffBT:IT‐4F and PhI‐ffBT:IT‐4F blend films show a strong IP (100) peak and a (010) scattering along the OOP direction, exhibiting predominant face‐on orientation characteristics, which should be beneficial for charge transport in the vertical direction of PSCs.^74^ According to the Scherrer's equation,^75^, ^76^ the crystal coherence lengths (CCLs) based on the OOP (010) π–π scattering peaks were calculated to be 34.96 and 28.55 Å for ffPhI‐ffBT:IT‐4F and PhI‐ffBT:IT‐4F films under the optimized device fabrication conditions. Although ffPhI‐ffBT:IT‐4F shows the slightly higher CCL value, the shorter π–π stacking distance was measured for PhI‐ffBT:IT‐4F (*q*_z_ = 1.75 Å^−1^, *d*‐spacing = 3.59 Å) with thermal treatments, compared to ffPhI‐ffBT:IT‐4F (*q*_z_ = 1.72 Å^−1^, *d*‐spacing = 3.65 Å) (Table S11, Supporting Information). The tighter π–π stacking in PhI‐ffBT:IT‐4F may improve vertical charge transport, which shows a good agreement with the SCLC mobility measurements (*µ*_h,SCLC_: 1.42 × 10^−3^ vs 5.61 × 10^−4^ cm^2^ V^−1^ s^−1^ for PhI‐ffBT:IT‐4F and ffPhI‐ffBT:IT‐4F, respectively), thus leading to the relatively higher FF and *J*_sc_ of the PhI‐ffBT‐based solar cells.

## 3 Conclusion

In summary, we have designed and synthesized two new D‐A_1_‐D‐A_2_ type donor polymers PhI‐ffBT and ffPhI‐ffBT based on the overlooked phthalimide as one of the acceptor units. The incorporation of second acceptor unit difluorobenzothiadiazole with a higher electron‐withdrawing capability (vs phthalimide) leads to optimized polymer optoelectronic properties. In comparison to all donor polymers with the state‐of‐the‐art performance in nonfullerene solar cells, the polymers reported here do not contain the widely used BDT as the donor unit, which can greatly enrich the materials library. More interestingly, these BDT‐free polymers show substantial charge carrier mobilities of 0.6–0.9 cm^2^ V^−1^ s^−1^ in organic thin‐film transistors, attributed to their more delocalized π‐conjugated systems and improved film crystallinity versus the BDT‐based polymers, which are beneficial to charge carrier transport and collection in solar cells. The fluorinated phthalimide‐based polymer ffPhI‐ffBT exhibits a slightly wider optical bandgap (1.77 eV) and a deeper‐positioned HOMO level (−5.63 eV) than the phthalimide‐based PhI‐ffBT (*E*_g_^opt^ = 1.75 eV, HOMO = −5.55 eV). The ffPhI‐ffBT:IT‐4F‐based PSCs show a high *V*_oc_ of 0.94 V and a relatively small energy loss of 0.58 eV with a PCE of 12.74%. Compared to the ffPhI‐ffBT‐based devices, the PhI‐ffBT‐based PSCs show a remarkable PCE of 13.31% with a higher *J*_sc_ of 19.41 mA cm^−2^ and a larger FF of 0.76, attributed to the improved film morphology and increased charge carrier mobilities. To the best of our knowledge, the PCE of 13.31% is the highest value except the most studied BDT‐based polymer semiconductors and is also the highest among all benzothiadiazole based polymers reported till today. These results demonstrate that both phthalimide and fluorinated phthalimide are excellent building blocks for enabling high mobility donor polymers with very promising device performance in nonfullerene PSCs and the D‐A_1_‐D‐A_2_ strategy is highly efficient for fine‐tuning polymer properties and enhancing their photovoltaic performances.

## Conflict of Interest

The authors declare no conflict of interest.

## Supporting information

Supplementary
