# Supplementary material for: Phthalimide‐Based High Mobility Polymer Semiconductors for Efficient Nonfullerene Solar Cells with Power Conversion Efficiencies over 13%
Source: Adv Sci (Weinh). 2018 Dec 12;6(2):1801743. doi: 10.1002/advs.201801743 (PMC6343056; doi:10.1002/advs.201801743)
Supplement: Supplementary file 1 — Supplementary [file ADVS-6-1801743-s001.pdf]

Copyright WILEY-VCH Verlag GmbH & Co. KGaA, 69469 Weinheim, Germany, 2018.

# Supporting Information

for *Adv. Sci.,* DOI: 10.1002/advs.201801743

# Phthalimide-Based High Mobility Polymer Semiconductors for Efficient Nonfullerene Solar Cells with Power Conversion Efficiencies over 13%

*Jianwei Yu, Peng Chen, Chang Woo Koh, Hang Wang, Kun Yang, Xin Zhou, Bin Liu, Qiaogan Liao, Jianhua Chen, Huiliang Sun, Han Young Woo, Shiming Zhang, and Xugang Guo**

Copyright WILEY-VCH Verlag GmbH & Co. KGaA, 69469 Weinheim, Germany, 2016.

# Supporting Information

**Phthalimide-Based High Mobility Polymer Semiconductors for Efficient Nonfullerene Solar Cells with Power Conversion Efficiencies over 13%**

Jianwei Yu, Peng Chen, Chang Woo Koh, Hang Wang, Kun Yang, Xin Zhou, Bin Liu, Qiaogan Liao, Jianhua Chen, Huiliang Sun, Han Young Woo, Shiming Zhang, Xugang Guo*

J. Yu, P. Chen, H. Wang, Dr. K. Yang, X. Zhou, B. Liu, Q. Liao, Dr. J. Chen, Dr. H. Sun, Prof. X. Guo
Department of Materials Science and Engineering and The Shenzhen Key Laboratory for Printed Organic Electronics, Southern University of Science and Technology, No. 1088, Xueyuan Road, Shenzhen, Guangdong, 518055, China
E-Mail: guoxg@sustc.edu.cn
J. Yu, H. Wang, Prof. S. Zhang
Key Laboratory of Flexible Electronics (KLOFE) & Institute of Advanced Materials (IAM), Jiangsu National Synergetic Innovation Center for Advanced Materials (SICAM), Nanjing Tech University (NanjingTech), 30 South Puzhu Road, Nanjing 211816, China
C. W. Koh, Prof. H. Y. Woo
Research Institute for Natural Sciences, Department of Chemistry, Korea University, Seoul 136-713, South Korea

## Table of Contents

1. Materials and Instruments.

2. Monomer and Polymer Synthesis.

3. Thermal, Electrochemical, and Optical Properties of Polymers.

4. DFT-Based Theoretical Calculations.

5. Fabrication and Characterization of Organic Thin-Film Transistors.

6. Fabrication and Characterization of Organic Solar Cells.

7. Polymer Film Morphology.

8. NMR Spectra of Compounds

## 1. Materials and Instruments.

All reagents and chemicals are commercially purchased and used without further purification unless otherwise stated. Anhydrous tetrahydrofuran and toluene are distilled from Na/benzophenone. The known monomer tributyl(4-(2-hexyldecyl)thiophen-2-yl)stannane[1] and 5,6-difluoro-4,7-diiodoisobenzofuran-1,3-dione[2] are prepared according to the published procedures. Monomer 4,7-dibromoisobenzofuran-1,3-dione and 5,6-difluoro-4,7-bis(5-(trimethylstannyl)thiophen-2-yl)benzo[c][1,2,5]thiadiazole were purchased from SunaTech Inc. (Suzhou, Jiangsu). All other reagents are used as received unless otherwise stated. All manipulations and reactions are performed under argon using the standard Schlenk line technique, and polymerizations are carried out on Initiator$^+$ Microwave Synthesizer (Biotage, Sweden). $^1$H and $^{13}$C spectra are recorded on a Bruker Ascend 500 MHz spectrometer. $^{19}$F spectra are recorded on a Bruker Ascend 400 MHz spectrometer. Chemical shifts were referenced to residual protio-solvent signals. C, H, N, S elemental analyses (EAs) of the polymers were performed at Nanjing Tech University (Nanjing, China). Molecular weights of the polymers were measured with a high-temperature GPC/SEC system (Agilent PL-GPC220) at 150 ºC versus polystyrene standard using 1,2,4-trichlorobenzene as the eluent. Thermogravimetric analysis (TGA) curves were collected using Mettler STAR$^e$ at a heating ramp of 10 ºC min$^{-1}$ in $N_2$ environment. Differential scanning calorimetry (DSC) curves were recorded with Mettler STAR$^e$ at a heating rate of 10 ºC min$^{-1}$ under $N_2$. Room temperature UV-vis spectra of the polymer solutions and

films were collected on a Shimadzu UV-3600 UV-VIS-NIR spectrophotometer. Temperature-dependent UV-vis absorption spectra of the polymer solutions were collected on Perkin Elmer Lambda 950 UV/VIS/NIR Spectrometer. Cyclic voltammetry (CV) measurements of the polymer films were conducted under argon atmosphere using a CHI760 Evoltammetric analyzer with 0.1 M tetra-n-butylammoniumhexafluorophosphate ($Bu_4NPF_6$) in acetonitrile ($CH_3CN$) as the supporting electrolyte. A platinum disk working electrode, a platinum wire counter electrode, and a silver wire reference electrode were employed, and ferrocene/ferroceniuum ($Fc/Fc^+$) redox couple was used as reference for all measurements. The scanning rate was 50 mV $s^{-1}$. Steady-state photoluminescence (PL) spectra were measured using a Horiba iHR320 spectrometer with the Andor Newton EMCCD detector. PL spectra were excited using a Coherent 532 CW laser. Atomic Force Microscopy (AFM) measurements were carried out using a Dimension Icon Scanning Probe Microscope (Asylum Research, MFP-3D-Stand Alone) in the tapping mode. Transmission Electron Microscopy (TEM) images were collected on Tecnai Spirit microscope (20 kV). For TEM samples, the films were first casted onto PEDOT:PSS covered substrates, and then were floated off in deionized $H_2O$ before being transferred onto TEM copper grids. Two-dimensional grazing incidence wide angle X-ray scattering (2D GIWAXS) measurements were performed at the PLS-II 9A U-SAXS beam line of Pohang Accelerator Laboratory, Korea.

## 2. Monomer and Polymer Synthesis.

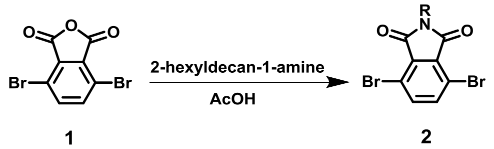


**4,7-dibromo-2-(2-hexyldecyl)isoindoline-1,3-dione (2).** Compound **1** (500 mg, 1.63 mmol), 2-hexyldecan-1-amine (506 mg, 1.63 mmol), and glacial acetic acid (5 mL) were combined and refluxed under $N_2$ for 6 h. After the acetic acid was removed under a reduced pressure, the crude product was purified via column chromatography on silica gel using dichloromethane:petroleum ether (1:1) as the eluent to give compound **2** as a white solid (776 mg, 90%). $^1$H NMR (500 MHz, $CDCl_3$), δ (ppm): 7.67 (s, 2H), 3.61 (m, 2H), 1.83 (m, 1H), 1.27 (m, 24H), 0.89 (m, 6H).

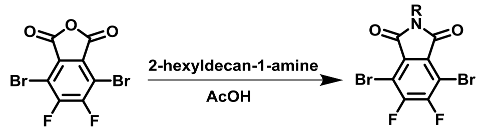


**5,6-difluoro-2-(2-hexyldecyl)-4,7-diiodoisoindoline-1,3-dione (4).** Compound **4** was synthesized following the same procedure employed in the synthesis of compound **2**, and the product was obtained as a white solid. (283 mg, 55%). $^1$H NMR (500 MHz, $CDCl_3$), δ (ppm): 3.61 (m, 2H), 1.83 (m, 1H), 1.32 (m, 24H), 0.92 (m, 6H).

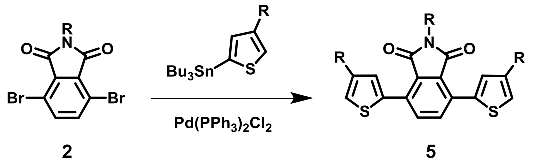


**2-(2-hexyldecyl)-4,7-bis(4-(2-hexyldecyl)thiophen-2-yl)isoindoline-1,3-dione (5).** An glass tube was charged with compound **2** (500 mg, 0.94 mmol),

tributyl(4-(2-hexyldecyl)thiophen-2-yl)stannane (1.69g, 2.83 mmol), and $Pd(PPh_3)_2Cl_2$ (63 mg, 0.09 mmol). The reaction tube and its contents were subjected to 3 pump/purge cycles with vacuum/argon, followed by the addition of anhydrous toluene (12 mL) via syringe. The tube was sealed under argon flow and then stirred at 130 ºC for 3 h under microwave irradiation. After the toluene was removed under a reduced pressure, the crude product was purified via column chromatography on silica gel using dichloromethane:petroleum ether (1:1) as the eluent to give compound **5** as a yellow oil (805 mg, 87%). $^1$H NMR (500 MHz, $CDCl_3$), δ (ppm): 7.76 (s, 2H), 7.62 (d, 2H), 7.04 (s, 2H), 3.60 (m, 2H), 2.64 (m, 4H), 1.92 (m, 1H), 1.68 (m, 2H), 1.37-1.26 (m, 72H), 0.96-0.86 (m, 18H).

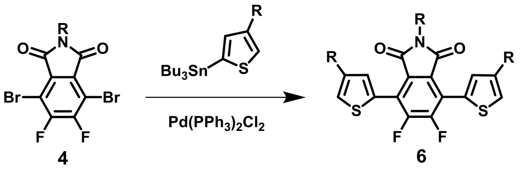


**5,6-difluoro-2-(2-hexyldecyl)-4,7-bis(4-(2-hexyldecyl)thiophen-2-yl)isoindoline-1,3-dione (6).** Compound **6** was synthesized following the same procedure employed in synthesis of compound **5**, and the product was obtained as a white solid. (527 mg, 83%). $^1$H NMR (500 MHz, $CDCl_3$), δ (ppm): 7.58 (s, 2H), 7.24 (s, 2H), 3.53 (m, 2H), 2.60 (m, 4H), 1.91 (m, 1H), 1.65 (m, 2H), 1.37-1.26 (m, 72H), 0.96-0.86 (m, 18H).

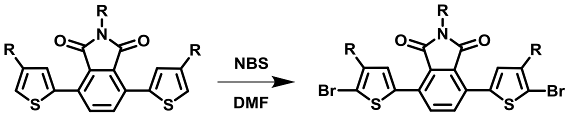


**4,7-bis(5-bromo-4-(2-hexyldecyl)thiophen-2-yl)-2-(2-hexyldecyl)isoindoline-1,3-dione (7).** Compound **5** (500 mg, 0.51 mmol) and NBS (182 mg, 1.02mmol) were

added into a glass bottle, followed by addition of 10 mL chloroform and 2 mL DMF. The mixture was stirred under room temperature overnight. Then, the mixture was extracted with dichloromethane, washed with water and then dried over anhydrous $MgSO_4$. After filtration, the solvent was removed under a reduced pressure. The crude product was purified via column chromatography on silica gel using ethyl dichloromethane:petroleum ether (1:10) as the eluent to give compound **7** as a yellow oil ( 542 mg, 93%). $^1$H NMR (500 MHz, $CDCl_3$), δ (ppm): 7.70 (s, 2H), 7.47 (s, 2H), 3.58 (m, 2H), 2.58 (m, 4H), 1.92 (m, 1H), 1.74 (m, 2H), 1.34-1.26 (m, 72H), 0.91-0.86 (m, 18H). $^{13}$C NMR (126 MHz, $CDCl_3$), δ (ppm): 167.34, 142.05, 136.30, 135.05, 131.67, 131.59, 127.67, 112.54, 42.56, 38.55, 36.82, 34.30, 33.34, 33.32, 31.96, 31.95, 31.92, 31.84, 31.47, 30.04, 29.71, 29.68, 29.58, 29.39, 29.33, 26.55, 26.27, 26.22, 22.72, 22.70, 22.67, 14.16, 14.12.

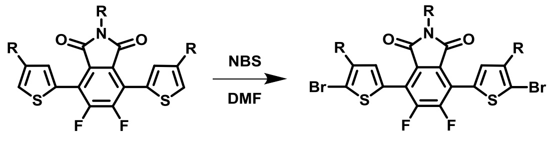


**4,7-bis(5-bromo-4-(2-hexyldecyl)thiophen-2-yl)-5,6-difluoro-2-(2-hexyldecyl)isoindoline-1,3-dione (8).** Compound **8** was synthesized following the same procedure employed in the synthesis of compound **7**, and the product was obtained as a white solid (625 mg, 90%).$^{19}$F NMR (400 MHz, $CDCl_3$) δ -125.65 (s, 2F). $^1$H NMR (500 MHz, $CDCl_3$), δ (ppm): 7.20 (s, 2H), 3.53 (m, 2H), 2.59 (m, 4H), 1.85 (m, 1H), 1.72 (m, 2H), 1.33-1.25 (m, 72H), 0.91-0.86 (m, 18H). $^{13}$C NMR (126 MHz, $CDCl_3$) δ (ppm): 165.54, 152.47-150.26, 141.39, 133.63, 126.50, 125.14, 122.35, 114.11, 42.89, 38.53, 36.82, 34.17, 33.35, 33.33, 31.96, 31.94, 31.81, 31.48, 30.02, 29.70, 29.67,

29.56, 29.39, 29.34, 26.59, 26.57, 26.25, 26.19, 22.72, 22.71, 22.67, 14.16, 14.13.

**General Procedure for Polymerizations via Stille Coupling for the Synthesis of Polymer PhI-ffBT and ffPhI-ffBT.** A flame dried glass tube was charged with two monomers (1.0 equiv each), $Pd_2(dba)_3$ (0.015 equiv), and $P(o\text{-}tolyl)_3$ (0.12 equiv). The tube and its contents were subjected to 3 pump/purge cycles with vacuum/argon, followed by the addition of anhydrous toluene (6-8 mL) via syringe. The tube was sealed under argon flow and then stirred at 80 ºC for 10 minutes, 110 °C for 10 minutes, and 140 °C for 1 h under microwave irradiation. Then, 0.1 mL of 2-(tributylstanny)thiophene was added and the reaction mixture was stirred under microwave irradiation at 140 °C for 0.5 h. Finally, 0.2 mL 2-bromothiophene was added and the reaction mixture was stirred at 140 °C for another 0.5 h. After cooling to room temperature, the reaction mixture was slowly dripped into 100 mL methanol containing 5 mL 12 N hydrochloric acid under vigorous stirring. After stirring for 4 h, the solid precipitate was transferred to a Soxhlet thimble. After drying, the crude product was subjected to sequential Soxhlet extraction with solvents and sequence depending on the solubility of the polymer. After final extraction, the polymer solution was concentrated to ~20 mL, and then dripped into 100 mL methanol under vigorous stirring. The polymer was collected by filtration and dried under a reduced pressure to afford a dark red solid as the product polymer.

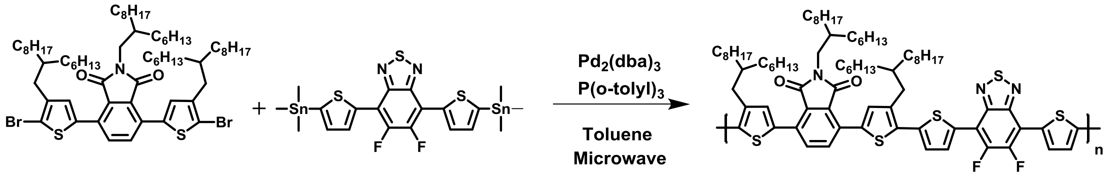

**PhI-ffBT.** The solvent sequence for Soxhlet extraction was methanol, acetone, hexane, dichloromethane, and chloroform. PhI-ffBT was obtained from chloroform fraction as a dark blue solid with a yield of 65%. $M_n$ = 36 kDa, PDI = 1.5. $^1$H NMR (400 MHz, $C_2D_2Cl_4$) 7.74 (s, 2H), 7.23-7.16 (m, 4H), 6.80 (m, 2H), 3.04 (m, 2H), 2.31 (m, 4H), 1.41 (m, 1H), 1.26 (m, 2H), 0.75-0.70 (m, 72H), 0.27 (m, 18H). Anal. Calcd for $C_{78}H_{107}F_2N_3O_2S_5$ (%): C, 71.13; H, 8.19; N, 3.19; S, 12.17. Found (%): C, 71.06; H, 8.02; N, 3.20; S, 12.61.

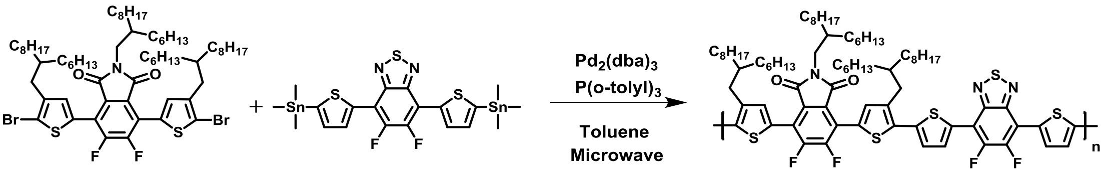


**ffPhI-ffBT.** The solvent sequence for Soxhlet extraction was methanol, acetone, hexane, dichloromethane, and chloroform. ffPhI-ffBT was obtained from chloroform fraction as a dark blue solid with a yield of 62%. $M_n$ =58 kDa, PDI = 1.2. $^1$H NMR (400 MHz, $C_2D_2Cl_4$) 7.75 (m, 2H), 7.86-7.82 (m, 4H), 3.99 (m, 2H), 2.33 (m, 4H), 1.33-1.27 (m, 3H), 0.82-0.70 (m, 72H), 0.28 (m, 18H). Anal. Calcd for $C_{78}H_{105}F_4N_3O_2S_5$ (%): C, 69.24; H, 7.82; N, 3.11; S, 11.85. Found (%): C, 69.23; H, 7.69; N, 3.12; S, 12.38.

## 3. Thermal, Electrochemical, and Optical Properties of Polymers.

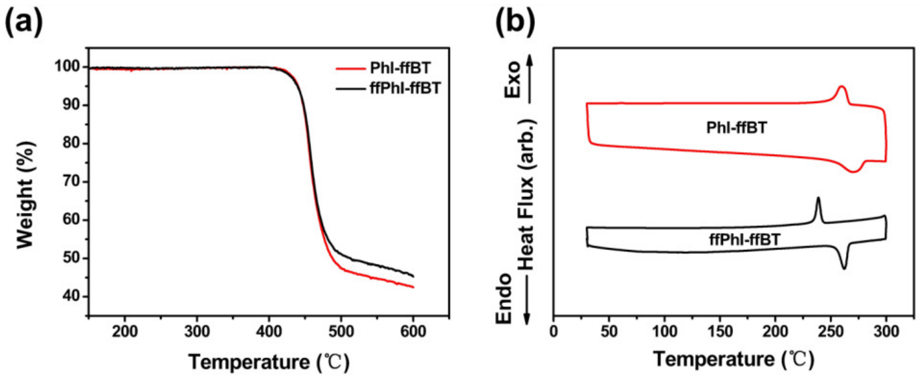


**Figure S1.** (a) TGA and (b) DSC curves of polymers PhI-ffBT and ffPhI-ffBT.

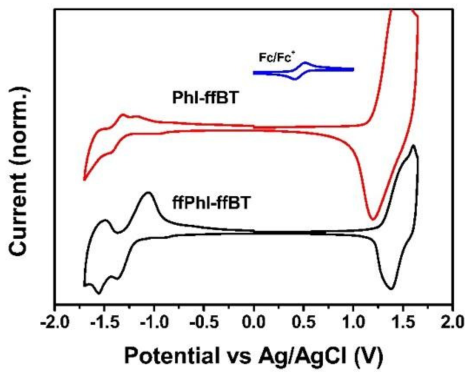


**Figure S2.** Cyclic voltammograms of PhI-ffBT and ffPhI-ffBT polymer films in 0.1 M $(n\text{-}Bu)_4N\cdot PF_6$ acetonitrile solution.

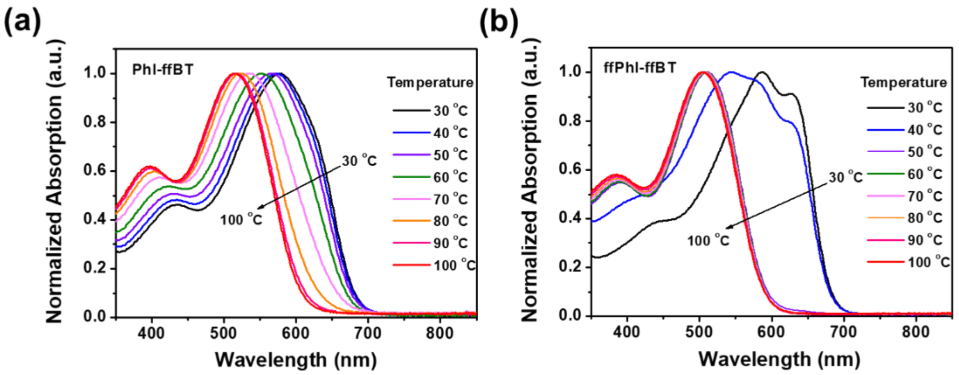


**Figure S3.** Temperature-dependent absorption spectra of polymers (a) PhI-ffBT and (b) ffPhI-ffBT in diluted chlorobenzene solutions.

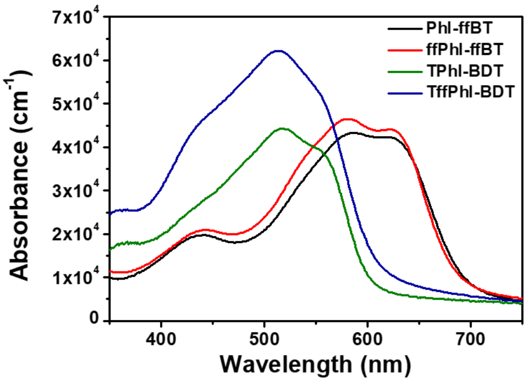


**Figure S4**. Optical absorption coefficients of PhI-ffBT, ffPhI-ffBT, TPhI-BDT, and TffPhI-BDT polymer films.

## 4. DFT-Based Theoretical Calculations.

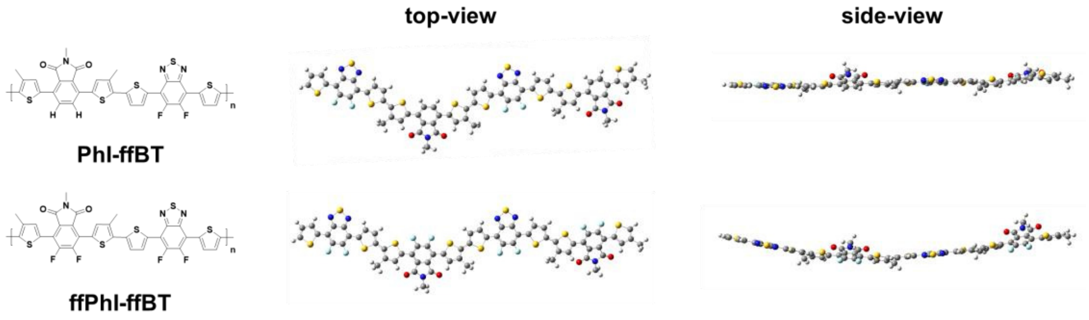


**Figure S5.** DFT calculated frontier molecular orbitals for the trimers of the repeating unit of polymers PhI-ffBT and ffPhI-ffBT under the optimized geometries.

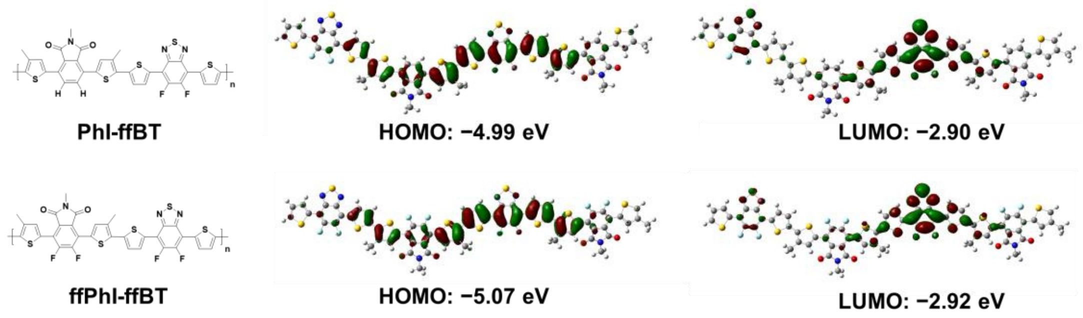


**Figure S6** Spatial distributions of FMOs of polymers PhI-ffBT and ffPhI-ffBT. The calculations were performed at the DFT//B3LYP/6-31G(d,p) level.

## 5. Fabrication and Characterization of Organic Thin-Film Transistors.

**Fabrication of Organic Thin-Film Transistors:** Top-gate/bottom-contact (TG/BC) organic field-effect transistors (OTFTs) were used for device fabrication and characterization. Source-drain electrodes (3 nm Cr and 30 nm Au) were patterned on borosilicate glass by photolithography, with a channel length of 10, 20, 50, or 100 μm and a channel width of 5 mm. The substrates were cleaned by sonication in acetone and isopropanol for 10 min each, followed by the UV-ozone treatment for 1 h. The polymer active layers were spin-coated from hot chlorobenzene solution (ffPhI-ffBT and PhI-ffBT, 5 mg $mL^{-1}$) or 1, 2-dichlorobenzene solutions (TPhI-BDT and TffPhI-BDT, 5 mg $mL^{-1}$), and then were thermally annealed at various temperatures for 20 min. The CYTOP dielectric layer was spin-coated (1500 rpm, 60 s) onto the semiconductor film from a diluted solution (CTL-809M:CTSOLV180 = 2:1, volume ratio, Asahi Glass Co., Ltd.), then annealed at 100 °C on a hot plate for 20 min. The thickness of the CYTOP layer is about 380 nm and an areal capacitance of 4.54 nF $cm^{-2}$ was used for mobility calculation. Finally, 50 nm Al was evaporated on top as the gate electrode to complete the device fabrication. The OTFT characterization was carried out inside a $N_2$-filled glove box with the Keithley S4200 semiconductor analyzer.

**Table S1.** Top-gate/bottom-contact (TG/BC) OTFT performance parameters of polymers PhI-ffBT and ffPhI-ffBT fabricated using chlorobenzene (CB) as the solvent and under various annealing temperature.

| Polymer | Solvent | $T_a$ (°C) | $\mu_{h,lin}$ ($cm^2\ V^{-1}\ s^{-1}$) [a] | $\mu_{h,sat}$ ($cm^2\ V^{-1}\ s^{-1}$) [a] | $V_T$ (V) [b] | $I_{on}/I_{off}$ [b] |
|---|---|---|---|---|---|---|
| PhI-ffBT | CB | 120 | 0.092 (0.087) | 0.52 (0.32) | -24 | $10^5$ |
| PhI-ffBT | | 160 | 0.21 (0.19) | 0.63 (0.51) | -33 | $10^5$ |
| PhI-ffBT | | 200 | 0.13 (0.11) | 0.60 (0.41) | -45 | $10^5$ |
| PhI-ffBT | | 240 | 0.17 (0.12) | 0.49 (0.42) | -48 | $10^5$ |
| ffPhI-ffBT | | 120 | 0.20 (0.19) | 0.86 (0.63) | -27 | $10^4$ |
| ffPhI-ffBT | | 160 | 0.23 (0.17) | 0.93 (0.72) | -40 | $10^4$ |
| ffPhI-ffBT | | 200 | 0.12 (0.10) | 0.44 (0.40) | -53 | $10^4$ |
| ffPhI-ffBT | | 240 | 0.15 (0.11) | 0.64 (0.47) | -57 | $10^4$ |

[a] Maximum mobility with average value from at least 5 devices are shown in parenthesis; [b] average values are shown in parenthesis.

**Table S2.** Top-gate/bottom-contact (TG/BC) OTFT performance parameters of BDT-based polymers TPhI-BDT and TffPhI-BDT fabricated using ortho-dichlororbenzene (o-DCB) as the solvent and under various annealing temperature.

| Polymer | Solvent | $T_a$ (°C) | $\mu_{h,lin}$ ($cm^2\ V^{-1}\ s^{-1}$) [a] | $\mu_{h,sat}$ ($cm^2\ V^{-1}\ s^{-1}$) [a] | $V_T$ (V) [b] | $I_{on}/I_{off}$ [b] |
|---|---|---|---|---|---|---|
| TPhI-BDT | o-DCB | 160 | $6.7\times10^{-3}$ ($6.4\times10^{-3}$) | $1.4\times10^{-2}$ ($1.1\times10^{-2}$) | -17 | $10^4$ |
| TPhI-BDT | | 200 | $1.1\times10^{-2}$ ($8.6\times10^{-3}$) | $1.4\times10^{-2}$ ($1.0\times10^{-2}$) | -12 | $10^4$ |
| TPhI-BDT | | 240 | $9.8\times10^{-3}$ ($8.6\times10^{-3}$) | $1.5\times10^{-2}$ ($1.3\times10^{-2}$) | -17 | $10^4$ |
| TffPhI-BDT | | 160 | $3.5\times10^{-3}$ ($3.0\times10^{-3}$) | $4.9\times10^{-3}$ ($3.9\times10^{-3}$) | -19 | $10^4$ |
| TffPhI-BDT | | 200 | $3.6\times10^{-3}$ ($2.7\times10^{-3}$) | $4.7\times10^{-3}$ ($4.2\times10^{-3}$) | -25 | $10^4$ |
| TffPhI-BDT | | 240 | $3.4\times10^{-3}$ ($2.7\times10^{-3}$) | $6.4\times10^{-3}$ ($5.7\times10^{-3}$) | -20 | $10^4$ |

[a] Maximum mobilities with average values from at least 5 devices are shown in parenthesis; [b] average values are shown in parenthesis.

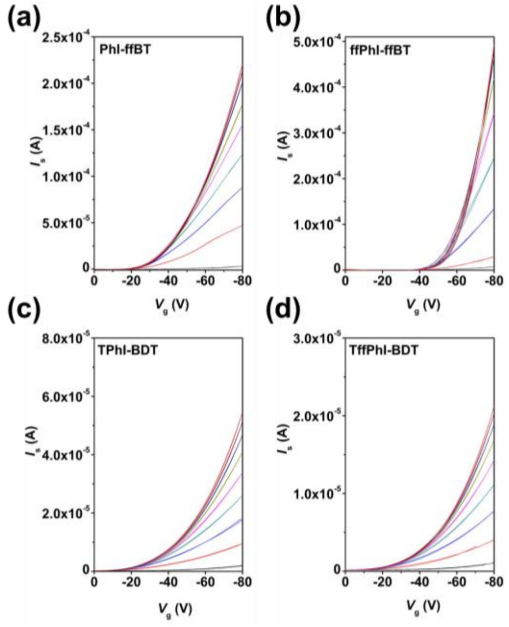


**Figure S7.** Top-gate/bottom-contact (TG/BC) OTFT transfer characteristics of polymer (a) PhI-ffBT; (b) ffPhI-ffBT; (c) TPhI-BDT; and (d) TffPhI-BDT. The gate voltage range is 0 to −80 V and -80 to 0 V with a 10 V interval in the plots.

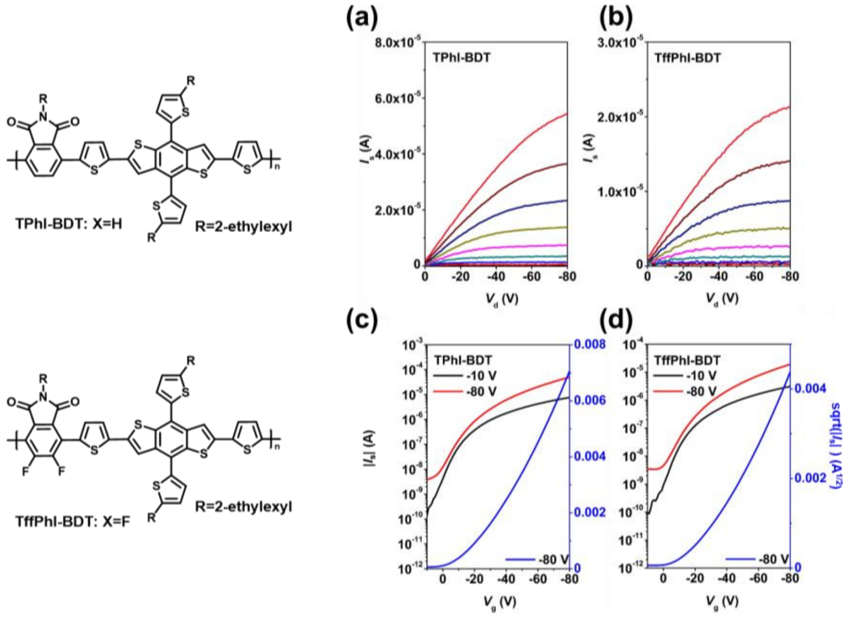


**Figure S8.** Top-gate/bottom-contact (TG/BC) OTFT (a, b) output and (c ,d) transfer characteristics of benzo[1,2-b:4,5-b′]dithiophene-based polymers (a, c) TPhI-BDT (L = 10 μm, W = 5 mm) and (b, d) TffPhI-BDT (L = 10 μm, W = 5 mm). The gate

voltage range is 0 to −80 V with −10 V intervals in the output plots. The polymer structures are shown in left.

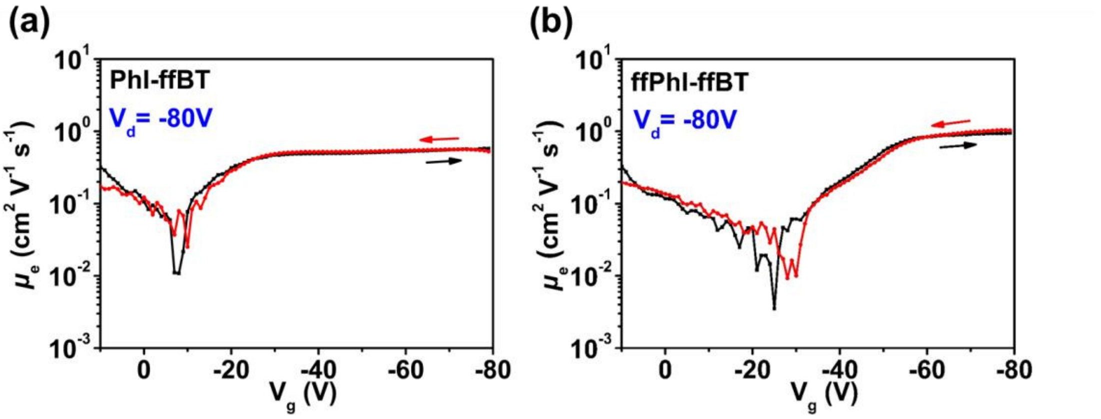


**Figure S9.** Mobility versus the gate voltage plot of polymers (a) PhI-ffBT and (b) ffPhI-ffBT based OTFTs.

## 6. Fabrication and Characterization of Polymer Solar Cells.

**Fabrication of Polymer Solar Cells with a Conventional Structure:** Pre-patterned Indium tin oxide (ITO)-coated glass substrates with a sheet resistance of ~12 Ω sq$^{-1}$ were used as the substrates, which were cleaned by sequential ultra-sonication in soap water, deionized water, acetone, and isopropanol for 15 min each, followed by UV-ozone treatment for 15 min. PEDOT:PSS (Clevios P VP A1 4083) was spin-coated onto the UV-ozone treated ITO substrates at 3000 rpm for 20 s to form a ~30 nm film, followed by thermal annealing at 150 °C for 15 min in air, The PEDOT:PSS-coated ITO substrates were then transferred into a $N_2$-filled glove box for subsequent steps. For ffPhI-ffBT donor polymer, the polymer:IT-4F (1.2:1 weight ratio) active layer solutions were prepared at a concentration of 10 mg mL$^{-1}$ in chlorobenzene (CB) with 0.6% volume ratio of 1,8-diiodooctane (DIO) as the

processing additive. The solutions were stirred and heated at 80 °C overnight to be completely dissolved. The ITO substrates were pre-heated on a hot plate at 80 °C before spin-coating the active layers at 1500 rpm. For PhI-ffBT, the polymer:IT-4F (1.2:1 weight ratio) active layer solution was prepared at a concentration of 7 mg $mL^{-1}$ (for the donor polymer) in chloroform (CF) with 0.6% volume ratio of DIO as the additive. The active layer solution was stirred at room temperature for 3 h before spin-coating at 4500 rpm. The spin-coated films were all vacuumed for 8 h to remove the high boiling point DIO additives. Before spin-coating the electron transporting layer, all active layers were thermally annealed at 125 °C for 5 min. Finally, 5 nm perylene diimide functionalized with amino N-oxide (PDINO)[3] (1.5 mg $mL^{-1}$ in ethanol) was spin-coated at 3000 rpm for 25 s on the active layer followed by the deposition of 100 nm Al cathode (area 4.5 $mm^2$ defined by the metal shadow mask) under a high vacuum (3E−6 Torr) using thermal evaporation.

**Fabrication of Polymer Solar Cells with Inverted Structure:** ITO-coated glass substrates with a sheet resistance of ~12 Ω $sq^{-1}$ were cleaned by the same procedures used for conventional cells. ZnO interfacial layer was spin-coated onto the ITO-coated glass substrates (3000 rpm, 30 s) and then baked in air at 200 °C for 30 min. The ZnO-coated substrates were brought into a $N_2$-filled glove box for the following steps. The optimal active layer preparation conditions established on the fabrication of conventional solar cells were used for the inverted solar cells. The spin-coated films were all vacuumed for 8 h to remove the high boiling point DIO

additives. Finally, $MoO_3$ (10 nm) and Ag (100 nm, area 4.5 $mm^2$ defined by the metal shadow mask) were subsequently deposited atop as the hole injection layer and top electrode in high vacuum (3E−6 Torr), respectively.

**Characterization of Polymer Solar Cells.** For polymer solar cell characterization, all current-voltage (J−V) characteristics of the devices were measured under simulated AM1.5G irradiation (100 mW/$cm^2$) using a Xe lamp-based SS-F5-3A Solar Simulator (Enli Technology, Inc.). A Xe lamp equipped with an AM1.5G filter was used as the white light source. The light intensity was controlled with an NREL-calibrated Si solar cell with a KG-5 filter. The external quantum efficiency (EQE) was measured by a QE-R3011 measurement system (Enli Technology, Inc.).

**Table S3.** Device performance parameters of conventional polymer solar cells (PSCs) using various amount of 1,8-diiodooctane (DIO) additives. (polymer:IT-4F = 1.2:1; concentration: 7 mg $mL^{-1}$ for PhI-ffBT and 10 mg $mL^{-1}$ for ffPhI-ffBT; solvent: CF for PhI-ffBT and CB for ffPhI-ffBT; active area: 4.5 $mm^2$)

| Polymer | Additive | $V_{oc}$ [a] (V) | $J_{sc}$ [a] (mA $cm^{-2}$) | FF [a] | PCE [a] (%) |
|---|---|---|---|---|---|
| PhI-ffBT | w/o | 0.91 (0.91 ± 0.01) | 18.51 (18.30 ± 0.13) | 0.62 (0.61 ± 0.01) | 10.41 (10.30 ± 0.10) |
| | 0.3% DIO | 0.90 (0.91 ± 0.01) | 18.32 (18.23 ± 0.14) | 0.70 (0.68 ± 0.01) | 11.50 (11.27 ± 0.14) |
| | 0.6% DIO | 0.91 (0.90 ± 0.01) | 19.41 (19.08 ± 0.27) | 0.76 (0.75 ± 0.01) | 13.31 (12.92 ± 0.23) |
| | 1.0% DIO | 0.88 (0.87 ± 0.004) | 15.86 (14.94 ± 1.07) | 0.63 (0.61 ± 0.02) | 8.88 (7.97 ± 0.66) |
| ffPhI-ffBT | w/o | 0.94 (0.94 ± 0.004) | 15.45 (14.82 ± 0.99) | 0.48 (0.48 ± 0.01) | 6.87 (6.68 ± 0.25) |
| | 0.3% DIO | 0.94 (0.94 ± 0.004) | 18.28 (18.24 ± 0.83) | 0.70 (0.69 ± 0.02) | 12.15 (11.80 ± 0.35) |
| | 0.6% DIO | 0.94 (0.94 ± 0.003) | 19.01 (18.84 ± 0.18) | 0.71 (0.71 ± 0.01) | 12.74 (12.42 ± 0.18) |
| | 1.0% DIO | 0.93 (0.93 ± 0.006) | 17.89 (17.63 ± 0.59) | 0.68 (0.02 ± 0.02) | 11.37 (10.79 ± 0.39) |

[a] The average values and standard deviations of the device parameters based on 10 devices are shown in parenthesis.

**Table S4.** Device performance parameters of conventional PSCs using different solvents. (polymer:IT-4F = 1.2:1; concentration: 7 mg $mL^{-1}$ for PhI-ffBT and 10 mg $mL^{-1}$ for ffPhI-ffBT; 0.6% DIO, volume percentage; active area: 4.5 $mm^2$)

| Polymer | Solvent | $V_{oc}$ [a] (V) | $J_{sc}$ [a] (mA $cm^{-2}$) | FF [a] | PCE [a] (%) |
|---|---|---|---|---|---|
| PhI-ffBT | CB | 0.92 (0.92 ± 0.004) | 16.69 (16.55 ± 0.22) | 0.61 (0.59 ± 0.02) | 9.40 (9.05 ± 0.35) |
| | CF | 0.91 (0.90 ± 0.01) | 19.41 (19.08 ± 0.27) | 0.76 (0.75 ± 0.01) | 13.31 (12.92 ± 0.23) |
| ffPhI-ffBT | o-DCB | 0.94 (0.94 ± 0.003) | 17.77 (17.65 ± 0.13) | 0.68 (0.68 ± 0.008) | 11.40 (11.20 ± 0.13) |
| | CB | 0.94 (0.94 ± 0.003) | 19.01 (18.84 ± 0.18) | 0.71 (0.71 ± 0.01) | 12.74 (12.42 ± 0.18) |

[a] The average values and standard deviations of the device parameters based on 10 devices are shown in

parenthesis.

**Table S5.** Device performance parameters of conventional PSCs using various polymer:IT-4F ratios. (concentration: 7 mg mL$^{-1}$ for PhI-ffBT and 10 mg mL$^{-1}$ for ffPhI-ffBT; solvent: CF for PhI-ffBT and CB for ffPhI-ffBT; 0.6% DIO, volume percentage; active area: 4.5 mm$^2$)

| Polymer | Polymer:IT-4F (w:w) | $V_{oc}$ [a] (V) | $J_{sc}$ [a] (mA cm$^{-2}$) | FF [a] | PCE [a] (%) |
|---|---|---|---|---|---|
| PhI-ffBT | 1.2:1 | 0.91 (0.90 ± 0.01) | 19.41 (19.08 ± 0.27) | 0.76 (0.75 ± 0.01) | 13.31 (12.92 ± 0.23) |
| | 1:1 | 0.91 (0.90 ± 0.01) | 18.32 (18.73 ± 0.39) | 0.73 (0.70 ± 0.02) | 12.17 (11.84 ± 0.17) |
| | 1:1.2 | 0.91 (0.91 ± 0.003) | 16.88 (16.73 ± 0.30) | 0.74 (0.73 ± 0.01) | 11.41 (11.16 ± 0.18) |
| ffPhI-ffBT | 1.2:1 | 0.94 (0.94 ± 0.003) | 19.01 (18.84 ± 0.18) | 0.71 (0.71 ± 0.01) | 12.74 (12.42 ± 0.18) |
| | 1:1 | 0.94 (0.93 ± 0.003) | 19.00 (18.85 ± 0.27) | 0.71 (0.70 ± 0.01) | 12.62 (12.21 ± 0.20) |
| | 1:1.2 | 0.93 (0.93 ± 0.002) | 19.46 (19.12 ± 0.28) | 0.66 (0.66 ± 0.01) | 12.01 (11.81 ± 0.13) |

[a] The average values and standard deviations of the device parameters based on 10 devices are shown in parenthesis.

**Table S6.** Device performance parameters of conventional PSCs using different thermal annealing (TA) temperatures. (concentration: 7 mg mL$^{-1}$ for PhI-ffBT and 10 mg mL$^{-1}$ for ffPhI-ffBT; polymer:IT-4F = 1.2:1; solvent: CF for PhI-ffBT and CB for ffPhI-ffBT; 0.6% DIO, volume percentage, active area: 4.5 mm$^2$)

| Polymer | Annealing | $V_{oc}$ [a] (V) | $J_{sc}$ [a] (mA/cm$^2$) | FF [a] | PCE [a] (%) |
|---|---|---|---|---|---|
| PhI-ffBT | w/o | 0.91 (0.91 ± 0.01) | 18.51 (18.30 ± 0.13) | 0.62 (0.61 ± 0.01) | 10.41 (10.30 ± 0.10) |
| | 100℃ | 0.90 (0.90 ± 0.002) | 19.34 (19.01 ± 0.25) | 0.75 (0.75 ± 0.01) | 13.14 (12.83 ± 0.16) |
| | 125℃ | 0.91 (0.90 ± 0.01) | 19.41 (19.08 ± 0.27) | 0.76 (0.75 ± 0.01) | 13.31 (12.92 ± 0.23) |
| | 150℃ | 0.89 (0.89 ± 0.003) | 18.02 (17.99 ± 0.06) | 0.66 (0.65 ± 0.01) | 10.65 (10.44 ± 0.20) |
| ffPhI-ffBT | w/o | 0.94 (0.94 ± 0.004) | 15.45 (14.82 ± 0.99) | 0.48 (0.48 ± 0.01) | 6.87 (6.68 ± 0.25) |
| | 100℃ | 0.94 | 17.23 | 0.72 | 11.51 |

| | | | | |
|---|---|---|---|---|
| | (0.93 ± 0.002) | (16.68 ± 0.55) | (0.72 ± 0.01) | (11.20 ± 0.26) |
| 125℃ | 0.94<br>(0.94 ± 0.003) | 19.01<br>(18.84 ± 0.18) | 0.71<br>(0.71 ± 0.01) | 12.74<br>(12.42 ± 0.18) |
| 150℃ | 0.92<br>(0.92 ± 0.01) | 17.38<br>(16.70 ± 0.65) | 0.66<br>(0.64 ± 0.02) | 10.51<br>(9.81 ± 0.38) |

[a] The average values and standard deviations of the device parameters based on 10 devices are shown in parenthesis.

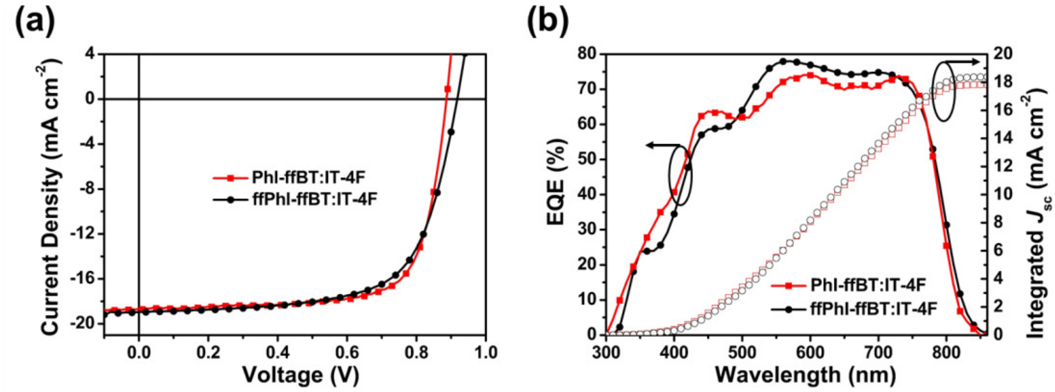


**Figure S10.** (a) J−V curves, (b) EQE spectra of the best-performing inverted polymer solar cells with the polymer:IT-4F blends as the active layers having an active area of 0.45 mm$^2$.

**Table S7.** Device performance parameters of PSCs using different device structures. (concentration: 7 mg mL$^{-1}$ for PhI-ffBT and 10 mg mL$^{-1}$ for ffPhI-ffBT; polymer:IT-4F = 1.2:1; solvent: CF for PhI-ffBT and CB for ffPhI-ffBT; 0.6% DIO, volume percentage; active area: 4.5 mm$^2$)

| Polymer | Device structure | $V_{oc}$ [c]<br>(V) | $J_{sc}$ [c]<br>(mA/cm$^2$) | FF [c] | PCE [c]<br>(%) |
|---|---|---|---|---|---|
| PhI-ffBT | Conventional [a] | 0.91<br>(0.90 ± 0.01) | 19.41<br>(19.08 ± 0.27) | 0.76<br>(0.75 ± 0.01) | 13.31<br>(12.92 ± 0.23) |
| | Inverted [b] | 0.89<br>(0.89 ± 0.01) | 18.66<br>(18.45 ± 0.28) | 0.73<br>(0.70 ± 0.01) | 12.14<br>(11.58 ± 0.37) |
| ffPhI-ffBT | Conventional [a] | 0.94<br>(0.94 ± 0.003) | 19.01<br>(18.84 ± 0.18) | 0.71<br>(0.71 ± 0.01) | 12.74<br>(12.42 ± 0.18) |
| | Inverted [b] | 0.92<br>(0.92 ± 0.001) | 18.94<br>(19.01 ± 0.22) | 0.67<br>(0.65 ± 0.01) | 11.60<br>(11.38 ± 0.14) |

[a] ITO/PEDOT:PSS/polymer:IT-4F/PDINO/Al; [b] ITO/ZnO/polymer:IT-4F/$MoO_3$/Ag; [c] The average values and standard deviations of the device parameters based on 10 devices are shown in parenthesis.

**Table S8.** Device performance parameters of conventional PSCs with different effective areas. (concentration: 7 mg mL$^{-1}$ for PhI-ffBT and 10 mg mL$^{-1}$ for ffPhI-ffBT; polymer:IT-4F = 1.2:1; solvent: CF for PhI-ffBT and CB for ffPhI-ffBT; 0.6% DIO, volume percentage)

| Polymer | Area ($mm^2$) | $V_{oc}$ [a] (V) | $J_{sc}$ [a] ($mA/cm^2$) | FF [a] | PCE [a] (%) |
|---|---|---|---|---|---|
| PhI-ffBT | 4.5 | 0.91 (0.90 ± 0.01) | 19.41 (19.08 ± 0.27) | 0.76 (0.75 ± 0.01) | 13.31 (12.92 ± 0.23) |
| | 10 | 0.90 (0.90 ± 0.006) | 19.73 (19.21 ± 0.29) | 0.73 (0.73 ± 0.01) | 12.93 (12.61 ± 0.14) |
| ffPhI-ffBT | 4.5 | 0.94 (0.94 ± 0.003) | 19.01 (18.84 ± 0.18) | 0.71 (0.71 ± 0.01) | 12.74 (12.42 ± 0.18) |
| | 10 | 0.94 (0.94 ± 0.003) | 19.31 (19.11 ± 0.12) | 0.67 (0.66 ± 0.01) | 12.24 (11.87 ± 0.24) |

[a] The average values and standard deviations of the device parameters based on 10 devices are shown in parenthesis.

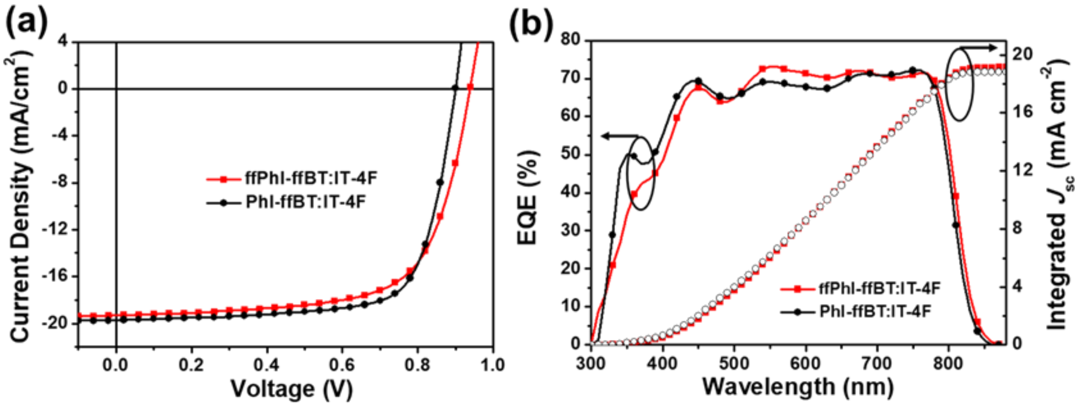


**Figure S11.** (a) *J*−*V* curves and (b) EQE spectra of the best-performing conventional polymer solar cells with the polymer:IT-4F blends as the active layers having an active area of 10 mm$^2$.

**SCLC Mobility Measurement.**

Mobility measurements using space charge limited current (SCLC) method were performed on both hole-only and electron-only devices. The structure of the hole-only device is ITO/PEDOT:PSS/polymer:IT-4F/$MoO_3$/Ag and the structure of the electron-only device is ITO/ZnO/polymer:IT-4F/PDINO/Al. The device fabrication conditions are the same as the OSCs described above. The SCLC mobility is extracted using Mott-Gurney equation[4]: $J = \frac{9}{8}\varepsilon_0\varepsilon_r\mu\frac{V^2}{d^3}$ where J is the current density, $\varepsilon_0$ is the vacuum permittivity ($8.85\times10^{-12}$ F $m^{-1}$), $\varepsilon_r$ is the relative dielectric constant of the active layer (assumed to be 3), $\mu$ is the hole or electron mobility, d is the thickness of the active layer, and V is the potential within the device given by $V = V_{appl}-V_{bi}$, where $V_{appl}$ is the voltage applied to the device and $V_{bi}$ is the build-in potential across the active layer and the electrode interface. $V_{bi}$ is taken to be zero for these hole-only and electron-only devices.

**Table S9.** The SCLC mobilities of the polymer:IT-4F blend films under the same conditions for the optimal solar cell fabrication after thermal annealing.

| Blend film | Thickness (nm) | $\mu_{e,SCLC}$ ($cm^2 V^{-1} s^{-1}$) | Thickness (nm) | $\mu_{h,SCLC}$ ($cm^2 V^{-1} s^{-1}$) |
|---|---|---|---|---|
| PhI-ffBT:IT-4F | 113 | $5.49\times10^{-4}$ | 120 | $1.42\times10^{-3}$ |
| ffPhI-ffBT:IT-4F | 170 | $2.21\times10^{-4}$ | 172 | $5.61\times10^{-4}$ |

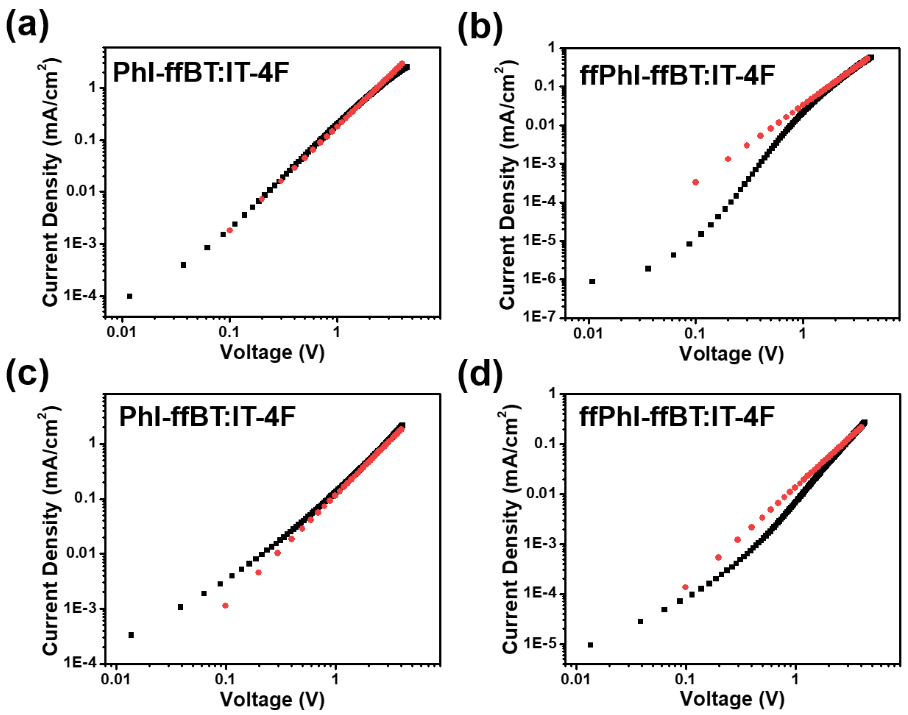


**Figure S12.** *J*–*V* characteristics of (a, b) the hole-only devices and (c, d) the electron-only devices. Experimental data was in black and the SCLC fitting was shown in red. For the electron-only device, the device structure is ITO/ZnO/polymer:IT-4F/PDINO/Al, while for hole-only device, the device structure is ITO/PEDOT:PSS/polymer:IT-4F/$MoO_3$/Ag.

**7. Polymer Film Morphology.**

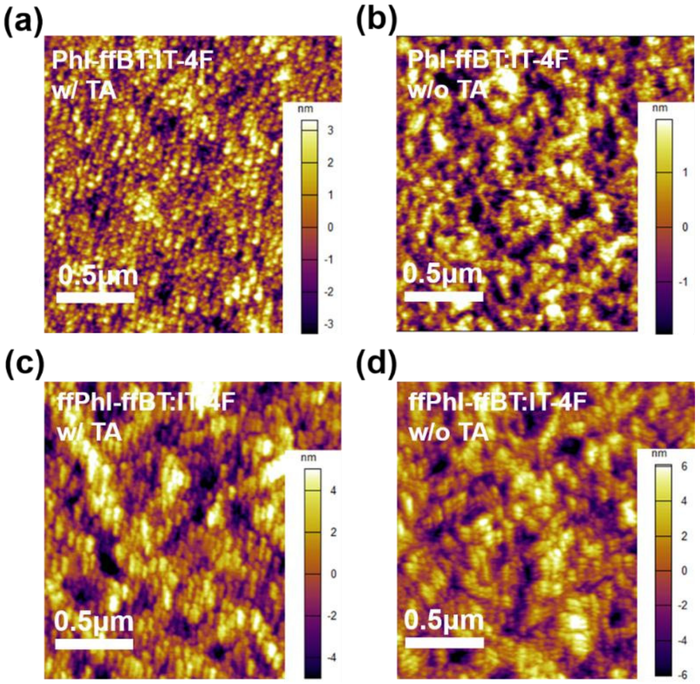


**Figure S13**. Tapping-mode AFM height images of polymer:IT-4F blend films: (a-b) PhI-ffBT:IT-4F and (c-d) ffPhI-ffBT:IT-4F

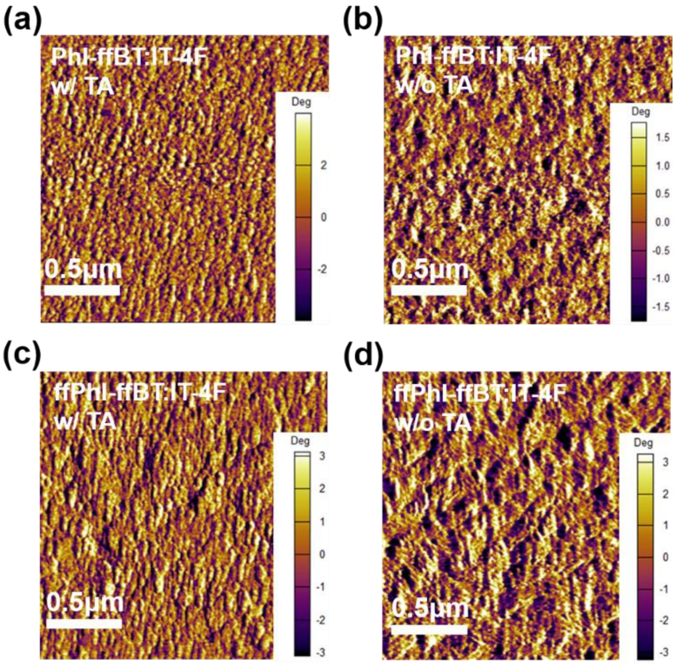


**Figure S14**. Tapping-mode AFM phase images of polymer:IT-4F blend films: (a-b) PhI-ffBT:IT-4F and (c-d) ffPhI-ffBT:IT-4F

**Table S10.** AFM measured root-mean-square (RMS) roughness values of the polymers:IT-4F blend films.

| Blend film | w/ TA (nm) | w/o TA (nm) |
|---|---|---|
| PhI-ffBT:IT-4F | 1.471 | 1.085 |
| ffPhI-ffBT:IT-4F | 2.270 | 2.333 |

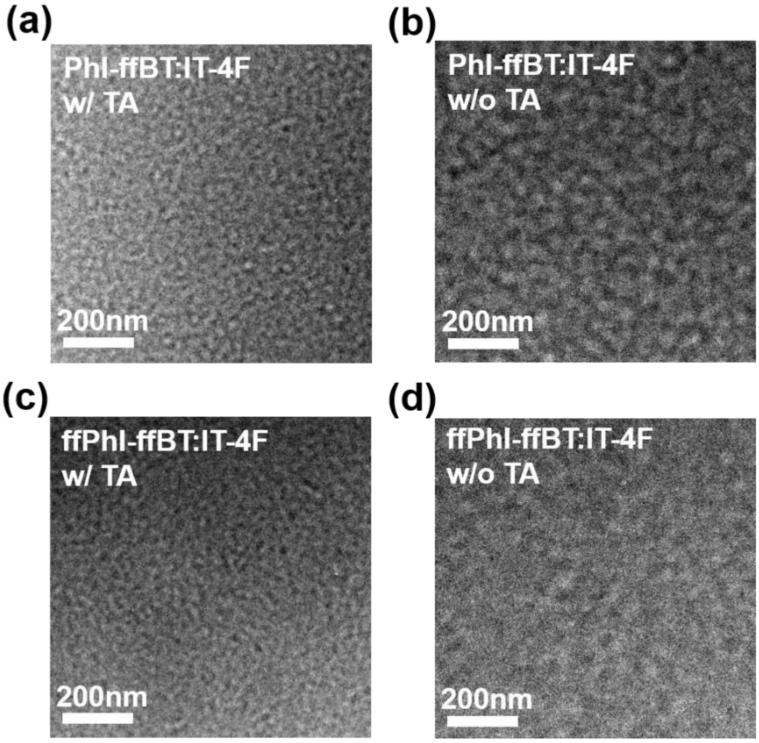


**Figure S15.** TEM images of polymer:IT-4F blend films: (a-b) PhI-ffBT:IT-4F and (c-d) ffPhI-ffBT:IT-4F

**Table S11**. Summary of packing parameters of polymers PhI-ffBT and ffPhI-ffBT neat and blend films.

| film | Plane | Direction | Lamellar spacing | | Direction | π-π stack | |
|---|---|---|---|---|---|---|---|
| | | | q (Å-1) | d-spacing (Å) | | q (Å-1) | d-spacing (Å) |
| PhI-ffBT | In | (100) | 0.2628 | 23.90 | | | |
| | Out | | | | (010) | 1.71 | 3.67 |
| PhI-ffBT :IT-4F | In | (100) | 0.283 | 22.19 | | | |
| | Out | | | | (010) | 1.717 | 3.66 |
| PhI-ffBT :IT-4F w/ TA | In | (100) | 0.3136 | 20.03 | | | |
| | Out | | | | (010) | 1.75 | 3.59 |
| ffPhI-ffBT | In | (100) | 0.2667 | 23.55 | (010) | 1.725 | 3.64 |
| | Out | (100) | 0.2633 | 23.85 | | | |
| | | (200) | 0.5406 | 23.23 | | | |
| | | (300) | 0.8312 | 22.67 | | | |
| ffPhI-ffBT :IT-4F | In | (100) | 0.2712 | 23.16 | | | |
| | | (200) | 0.5448 | 23.05 | | | |
| | Out | (100) | 0.2633 | 23.85 | (010) | 1.708 | 3.68 |
| | | (200) | 0.5384 | 23.33 | | | |
| ffPhI-ffBT :IT-4F w/ TA | In | (100) | 0.2734 | 22.97 | | | |
| | | (200) | 0.5603 | 22.42 | | | |
| | Out | (100) | 0.2633 | 23.85 | (010) | 1.722 | 3.65 |
| | | (200) | 0.5296 | 23.72 | | | |

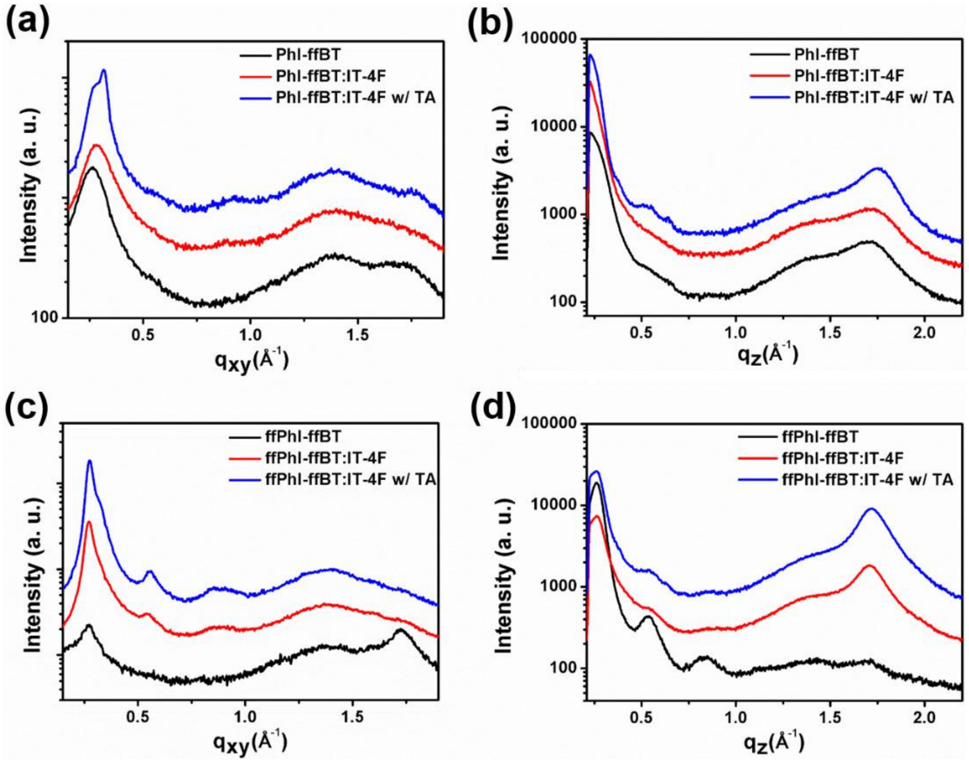


**Figure S16.** Out-of-plane ($q_z$) and in-plane ($q_{xy}$) line cuts of the GIWAXS for (a, b) PhI-ffBT-based films and (c, d) ffPhI-ffBT-based films.

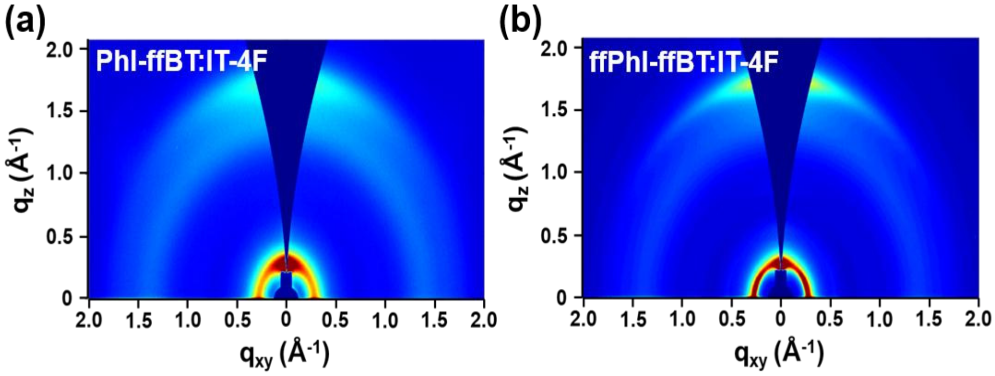


**Figure S17.** GIWAXS images of (a) PhI-ffBT:IT-4F blend film without thermal treatment and (b) ffPhI-ffBT:IT-4F blend film without thermal treatment.

## 8. NMR Spectra of Compounds.

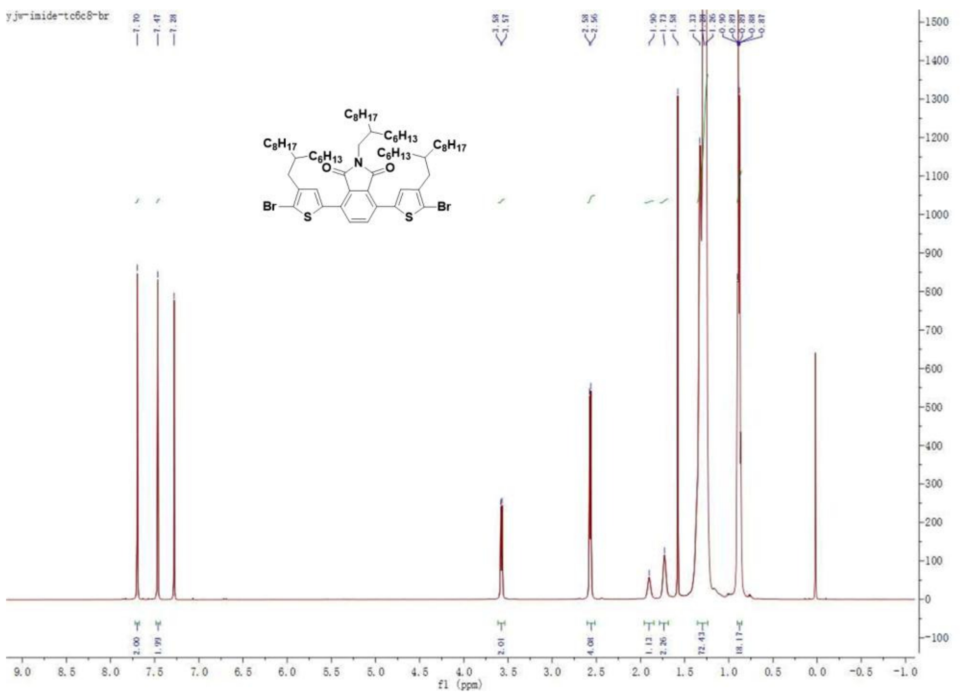


**Figure S18**. $^{1}H$ NMR spectrum of monomer **7**.

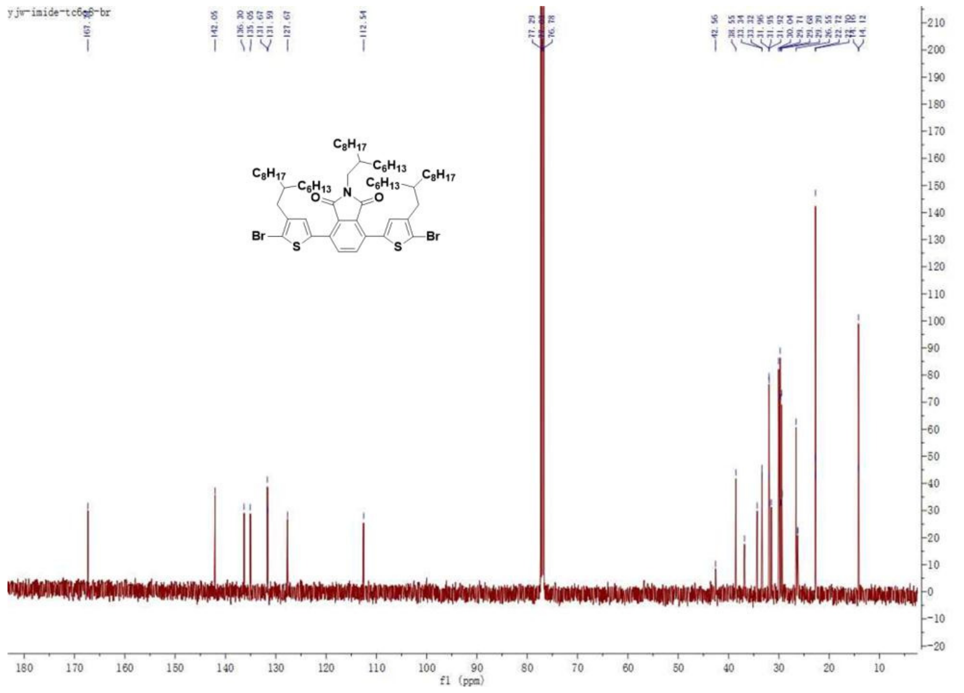


**Figure S19**. $^{13}C$ NMR spectrum of monomer **7**.

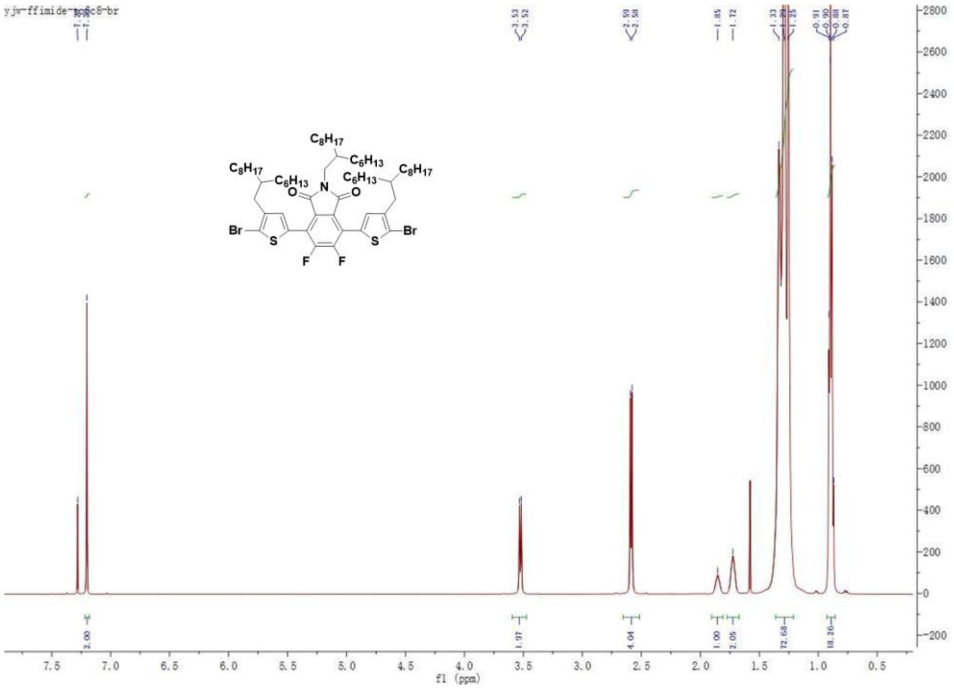

**Figure S20**. $^{1}$H NMR spectrum of monomer **8**.

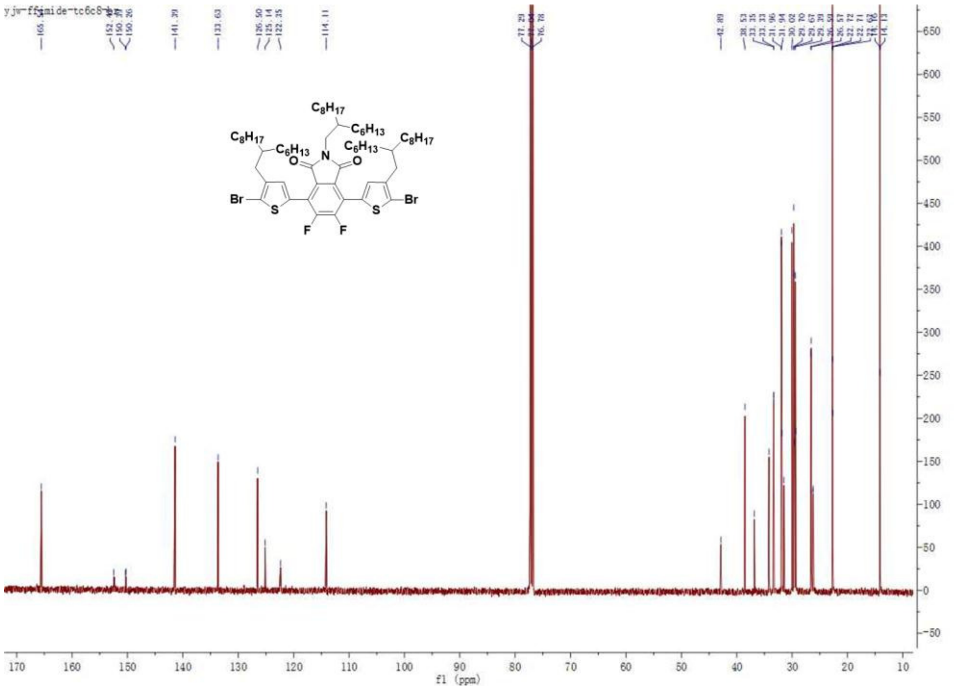

**Figure S21**. $^{13}$C NMR spectrum of monomer **8**.

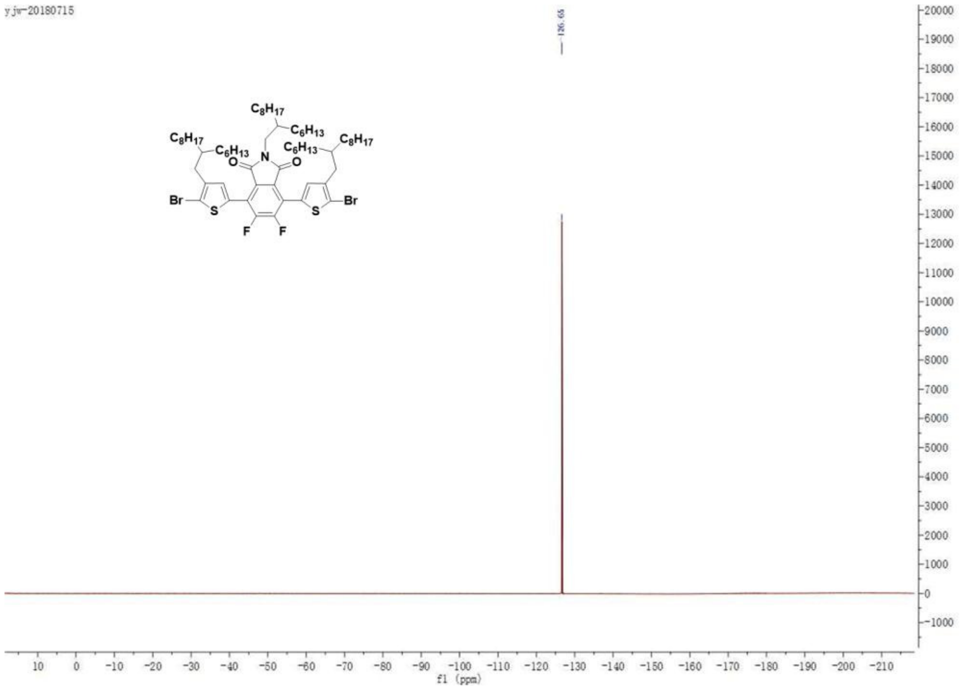

**Figure S22**. $^{19}$F NMR spectrum of monomer **8**.

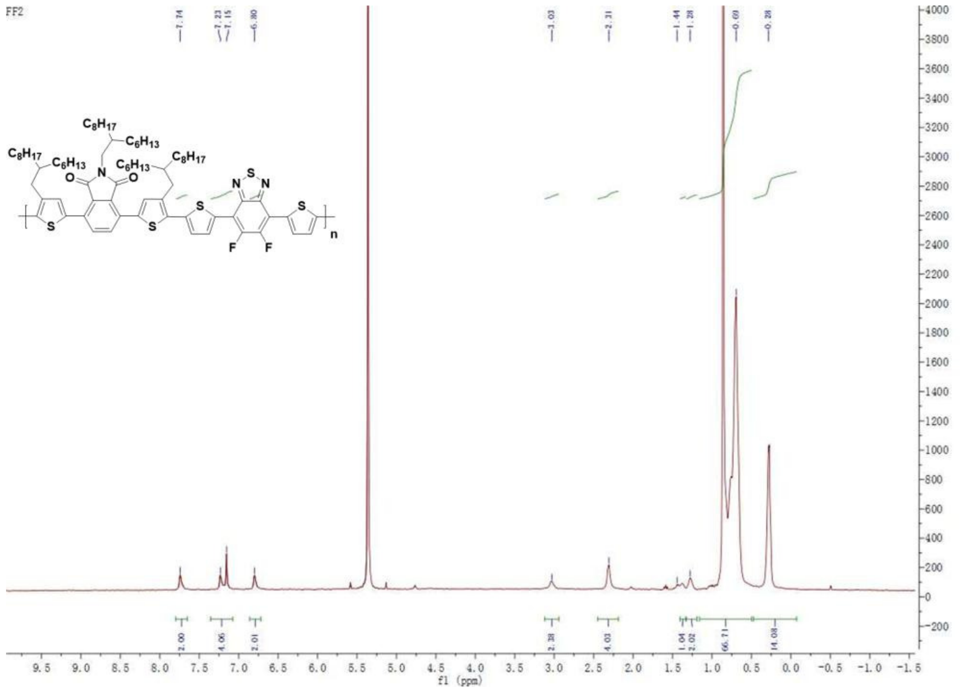

**Figure S23.** $^{1}$H NMR spectrum of polymer PhI-ffBT (80 °C, in $C_2D_2Cl_4$).

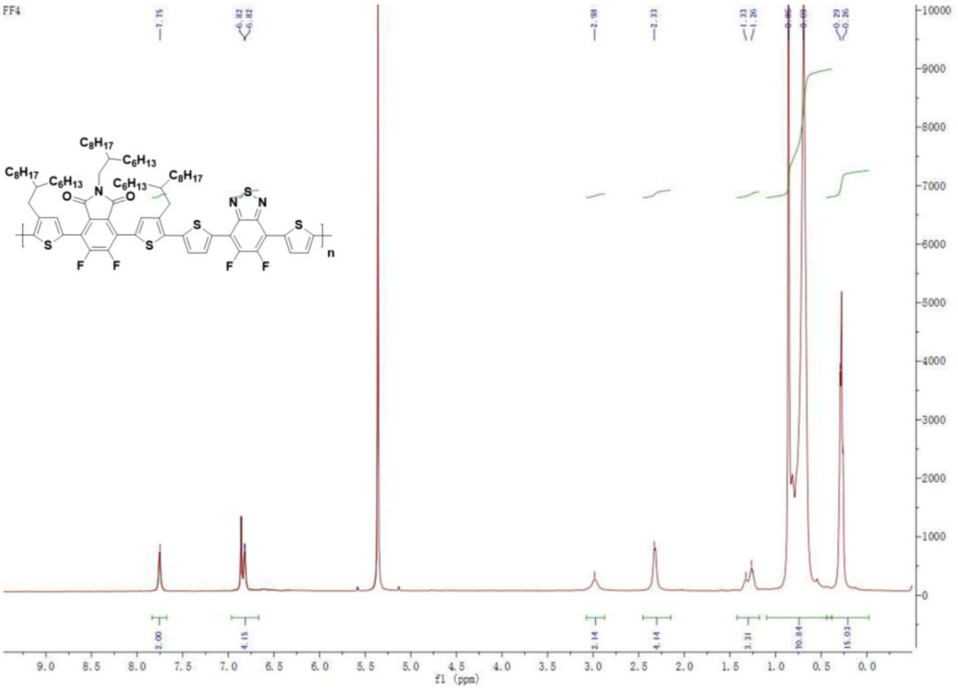

**Figure S24.** $^1$H NMR spectrum of polymer ffPhI-ffBT (80 °C, in $C_2D_2Cl_4$).

**References:**


[1] S. Shi, Y. Wang, M. A. Uddin, X. Zhou, H. Guo, Q. Liao, X. Zhu, X. Cheng, H. Y. Woo, X. Guo, Adv. Electron. Mater. **2017**, 3, 1700100.

[2] J. Yu, J. Yang, X. Zhou, S. Yu, Y. Tang, H. Wang, J. Chen, S. Zhang, X. Guo, Macromolecules **2017**, 50, 8928.

[3] Z.-G. Zhang, B. Qi, Z. Jin, D. Chi, Z. Qi, Y. Li, J. Wang, Energy Environ. Sci. **2014**, 7, 1966.

[4] P. Murgatroyd, J. Phys. D: Appl. Phys. **1970**, 3, 151.
